# Tracing the fate and transport of secondary plant metabolites in a laboratory mesocosm experiment by employing mass spectrometric imaging

**DOI:** 10.1007/s00216-017-0325-7

**Published:** 2017-03-29

**Authors:** Anna C. Crecelius, Beate Michalzik, Karin Potthast, Stefanie Meyer, Ulrich S. Schubert

**Affiliations:** 1 Laboratory of Organic and Macromolecular Chemistry (IOMC), Friedrich Schiller University Jena, Humboldtstrasse 10, 07743 Jena, Germany; 2 Jena Center for Soft Matter (JCSM), Friedrich Schiller University Jena, Philosophenweg 7, 07743 Jena, Germany; 3 Institute of Geography, Friedrich Schiller University Jena, Löbdergraben 32, 07743 Jena, Germany

**Keywords:** Laser desorption/ionization, Time-of-flight, Mass spectrometric imaging, Mesocosm, Secondary plant metabolites

## Abstract

Mass spectrometric imaging (MSI) has received considerable attention in recent years, since it allows the molecular mapping of various compound classes, such as proteins, peptides, glycans, secondary metabolites, lipids, and drugs in animal, human, or plant tissue sections. In the present study, the application of laser-based MSI analysis of secondary plant metabolites to monitor their transport from the grass leaves of *Dactylis glomerata*, over the crop of the grasshopper *Chorthippus dorsatus* to its excrements, and finally in the soil solution is described. This plant-herbivore-soil pathway was investigated under controlled conditions by using laboratory mesocosms. From six targeted secondary plant metabolites (dehydroquinic acid, quinic acid, apigenin, luteolin, tricin, and rosmarinic acid), only quinic acid, and dehydroquinic acid, an in-source-decay (ISD) product of quinic acid, could be traced in nearly all compartments. The tentative identification of secondary plant metabolites was performed by MS/MS analysis of methanol extracts prepared from the investigated compartments, in both the positive and negative ion mode, and subsequently compared with the results generated from the reference standards. Except for tricin, all secondary metabolites could be tentatively identified by this approach. Additional liquid-chromatography mass spectrometry (LC-MS) experiments were carried out to verify the MSI results and revealed the presence of quinic acid only in grass and chewed grass, whereas apigenin-hexoside-pentoside and luteolin-hexoisde-pentoside could be traced in the grasshopper body and excrement extracts. In summary, the MSI technique shows a trade-off between sensitivity and spatial resolution.

**Electronic supplementary material:**

The online version of this article (doi:10.1007/s00216-017-0325-7) contains supplementary material, which is available to authorized users.

**Graphical abstract:**
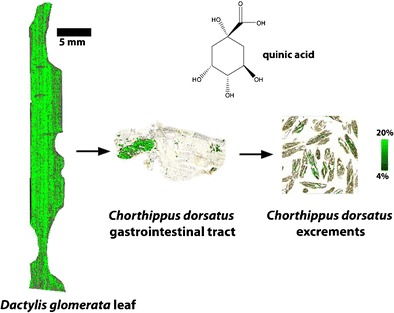
Monitoring quinic acid in a mesocosm experiment by mass spectrometric imaging (MSI).

## Introduction

In view of current climate change, more extreme weather phenomena are projected to occur, which, in turn, favor ecosystem disturbances such as mass outbreaks of herbivore insects [1, 2]. Due to their feeding and excreting activities, insect pests are known to facilitate the release of nutrients previously bound in plants, which have been found to be particularly important for changes in the rates of nutrient cycling due to the prompt availability to soil microbes and plants [3, 4]. Furthermore, recent studies point to herbivore-induced alterations in the timing and the quality of organic matter fractions reaching the forest floor as compared to organic matter in litterfall and the potential impact on belowground processes [5]. As a consequence, the knowledge of the effect of, e.g., herbivore feeding activity on the cycling of specific organic substances in an ecosystem and their use as biomarkers for tracing the source of dissolved organic matter (DOM) in soil is of great value.

To shade more light into the impact of insect feeding activity on the composition of DOM, a laboratory mesocosm experiment comprising the grass species *Dactylis glomerata* and the grasshopper species *Chorthippus dorsatus* was conducted. The idea was to follow the abundance and compositional change of organic substances along a plant-herbivore-excretions (feces)-soil solution pathway by the application of emerging visualization techniques, namely laser desorption/ionization time-of-flight mass spectrometric imaging (LDI-TOF MSI) and matrix-assisted laser desorption/ionization time-of-flight mass spectrometric imaging (MALDI-TOF MSI) [6, 7]. The latter technique requires the evaluation of suitable matrices for the analysis of secondary plant metabolites [8, 9], in particular when different objects (grass leaves, grasshopper, excrements) are analyzed.

The analysis of leaf surfaces by laser-based MSI techniques has been often described in recent literature [10, 11], even the quantification of plant surface metabolites [12]; however, the common grass species *D. glomerata* has up to now not been investigated. A few studies about the MALDI MSI analysis of insects, such as beetles [13], flies [14–16], and honeybees [17], can be found in the literature; however, locusts have only been imaged employing desorption electrospray ionization (DESI) to show the distribution of the pharmaceutical compound terfenadine and its metabolites [18].

In the current study, special emphasis was drawn to the role of secondary plant metabolites in organic matter cycling, since only very little knowledge is available so far [19]. In particular, the insect-mediated formation and fate of polyphenols from plant tissue via insect excretion to their ultimate mineralization is still uncertain. Polyphenols, thereby, are the most distributed class of secondary metabolites and, e.g., contribute to the plant color, defend the plants against herbivory, and increase the plants’ fitness [19, 20]. In many studies, phenolic compounds have been shown to function as allelochemicals, referring to an “interference mechanism in which live or dead plant materials including plant litter release chemicals which exert an effect (usually negative) on associated plants” [21]. For example, secondary metabolites inhibit both germination and growth of plants, thereby affecting the distribution patterns and composition of understory vegetation of forests [22, 23]; furthermore, they affect the growth of mycorrhizal fungi [24], inhibit feeding by various fungi [25], and potential herbivores [26, 27].

Four polyphenols (apigenin, luteolin, tricin, and rosmarinic acid) and two cyclic polyols (dehydroquinic acid and quinic acid) were evaluated as biomarkers to trace the source of DOM in soil, since they were easily detectable via MSI on the grass species *D. glomerata*. To the best of our knowledge, this is the first study on the application of spectrometric imaging techniques to track secondary metabolites in a model ecosystem along a cascade of different trophic levels.

## Materials and methods

### Chemicals

HPLC-grade methanol and water were purchased from VWR International (Dresden, Germany). Sucrose, trifluoro acetic acid, and the reference substances 3-dehydroquinic acid potassium salt, quinic acid, rosmaricic acid, tricin-5-glycoside, cryptochlorgenic acid, apigenin, apigenin-7-glycoside, luteolin, and luteolin-7-glycoside were supplied by Sigma-Aldrich (Steinheim, Germany). Gelatin was bought from Merck KGaA (Darmstadt, Germany). The matrices α-cyano-4-hydroxycinnamic acid (α-CHCA), 2,5-dihydroxybenzoic acid (DHB) were purchased from Bruker Daltonik GmbH (Bremen, Germany), and 1,5-diaminonaphthalene (1,5-DAN) from Sigma-Aldrich.

### Herbivory experiments

**Fig. 1:**
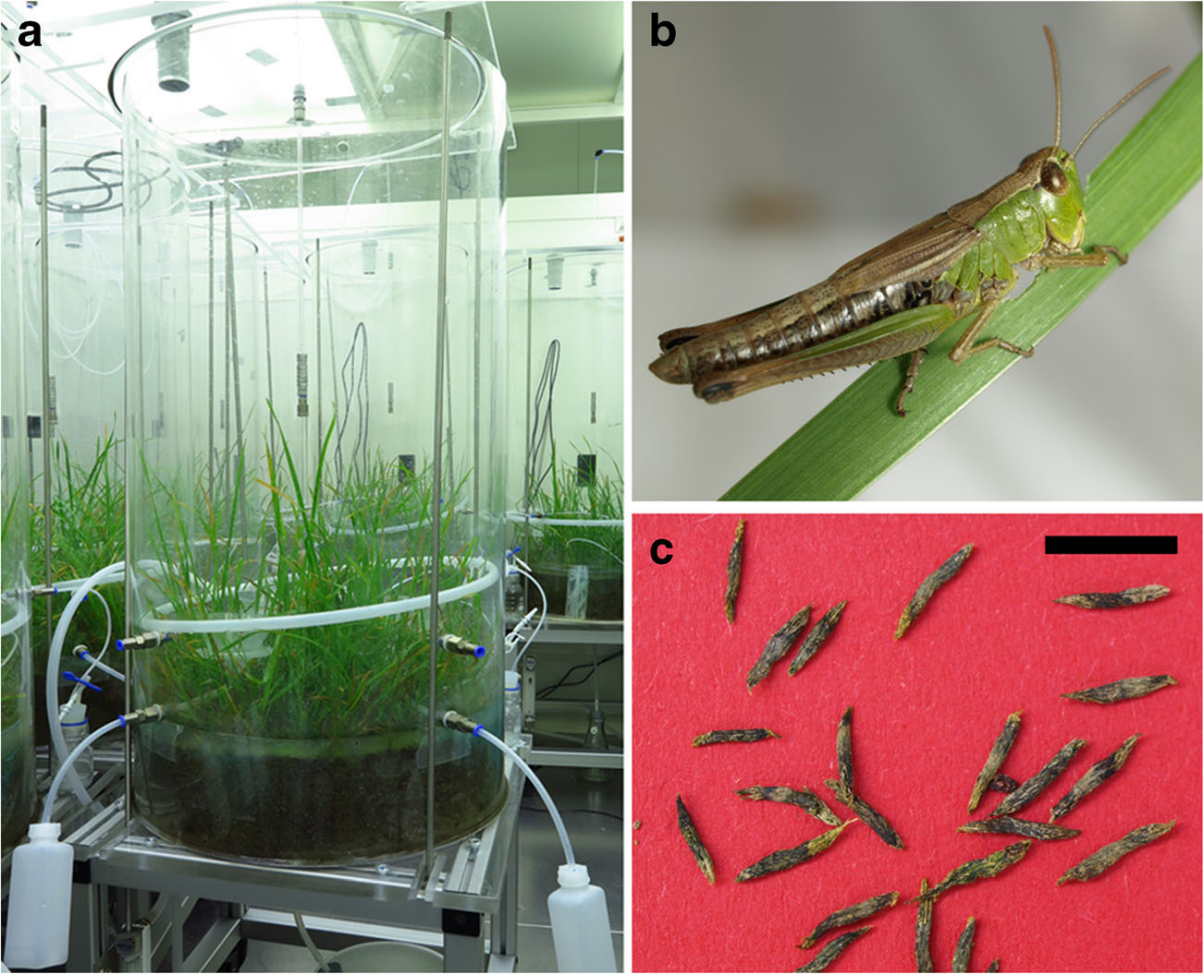
Laboratory mesocosm experiment: The photographs show **a** the mesocosms with the grown grass species *D. glomerata* in the climatic chamber; **b** a grasshopper from the species *C. dorsatus*; and **c** its feces. *Scale bar* 5 mm (**c**)

**Fig. 2:**
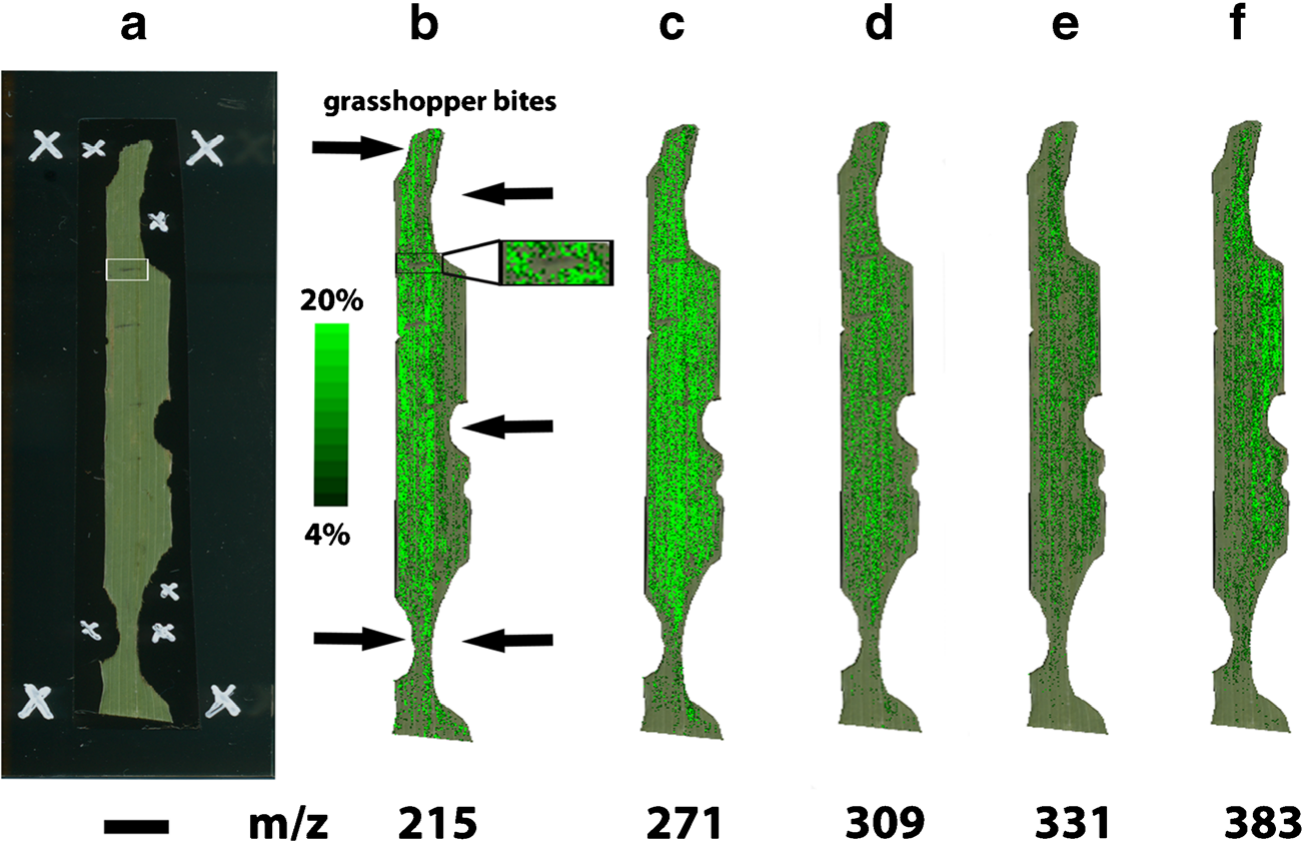
MALDI-TOF MSI analysis of a *D. glomerata* leaf. **a** Optical image taken before the matrix application, and ion images (*green color*) overlaid onto the optical image showing the distribution of **b** quinic acid (*m/z* 215), **c** apigenin (*m/z* 271), **d** luteolin (*m/z* 309), **e** tricin (*m/z* 331), and **f** rosmarinic acid (*m/z* 383) Note: The assignments of the secondary plant metabolites are tentative based on the MS and MS/MS data of reference standards, as presented in the ESM, and additional MS and MS/MS experiments of methanol extracts of *D. glomerata* leaves, as shown in Figs. 3 and 4, respectively. The ion images are normalized using the TIC. *Scale bar* 5 mm (**a**)

To trace the fate of secondary metabolites in a pasture ecosystem invaded by grasshoppers, mesocosm experiments with the common pasture grass *D. glomerata* and with homogenized pasture soil (0 to 16 cm, sieved to 6 mm) were conducted under controlled environmental conditions in a climatic chamber. To simulate a well-established pasture, seeds of *D. glomerata* were distributed homogeneously with a hole template on the soil surface of the mesocosms (12 cm soil depth, 50 cm in diameter, about 4 seeds/dm^2^) 1 year before the experiments started. During growth, the grass was cultivated in a climatic chamber (15 °C, automated weekly watering), as presented in Fig. 1a. In April 2015, the grass was cut once to a height of 4 cm to simulate grazing. Before the start of the herbivory experiment, grasshoppers, *C. dorsatus*, were caught on a grassland next to Jena. In October 2015, the herbivory experiment started when 20 grasshoppers were housed in one mesocosm and subjected to a 8-h dark and 16-h light cycle for 5 days, as presented in Fig. 1. In parallel mesocosms without grasshopper amendment were used as control. The grasshoppers had constant access to the pasture grass. At the end of the experiment, chewed grass leaves (see Fig. 2a), grasshoppers (see Fig. 1b), feces (see Fig. 1c), and soil solutions (cold water extracts of soil samples: control and grasshopper mesocosms) were taken to conduct the visualization of polyphenols and cyclic polyols in these compartments. The respective sample preparations and applications for MSI investigations are given below:

#### Grass leaves

Fresh chewed leaves were fixed on double-sided conductive tape (65 mm × 20 mm, Plano GmbH, Wetzlar, Germany) and attached to indium tin oxide (ITO)-coated glass slides (Bruker Daltonik GmbH, Bremen, Germany). Each grass leaf was mounted on one ITO-coated glass slide. Afterwards, teaching points were added with a white paint marker and the slide was scanned with an Epson perfection v700 photo scanner (Epson Europe B. V., Amsterdam, Netherlands). Figure 2a shows such a photographic image. The matrix was either sprayed on the grass leaf using an ImagePrep device (Bruker Daltonik GmbH) following the manufacture’s protocol or with an air-brush device (Revell GmbH, Bünde, Germany). For the latter method, the ITO-coated glass slide with the sample was mounted upright 25 cm from the air-brush outlet and 5 s of spraying was alternated with 1 min of air-drying. Between 10 and 15 cycles of matrix application produced a homogeneous layer of matrix crystals. Afterwards, the sample was stored in a desiccator for approximately 1 h. The positive ion mode with 100 μm spatial resolution and within a mass range of *m/z* 200 to 1500 was selected for MALDI-TOF MSI analysis.

#### Grasshoppers

At the end of the experiments, the gastrointestinal tract was extracted from a grasshopper body that was emerged for only 1 s in liquid nitrogen, as shown in Fig. 5a, b. It was quite difficult to extract the gastrointestinal tract intact, hence female grasshoppers were selected, since their body is greater compared to the male ones. The extracted tissue was shock frozen in liquid nitrogen and stored in a −80 °C freezer until further analysis. A single grasshopper gastrointestinal tract was mounted with a droplet of phosphate-buffered saline (PBS) on the sample holder in a CM 1860 cryostat (Leica Biosystems, Nussloch, Germany). Twelve micrometers thick sections were cut at −21 °C and thaw-mounted on conductive ITO-coated glass slides. Between one and two sections were placed on a single ITO-coated glass slide and stored in a desiccator for approximately 1 h. LDI-TOF MSI experiments of the gastrointestinal tract of a grasshopper were performed in positive ion mode with 50 μm spatial resolution and within a mass range of *m/z* 0 to 1000.

#### Excrements

One-day-old feces were stored at room temperature in a glass vial until further analysis the next day. The feces were embedded in gelatin (10% *w*/*v*) using a disposable specimen mold (Ø 22 mm, Plano GmbH, Wetzlar, Germany), as shown in Fig. 6a. Twelve micrometers thick sections were cut in a cryostat using a temperature of −21 °C (see Fig. 6b) and thaw-mounted on conductive ITO-coated glass slides. One section was fitting on one ITO-coated glass slide and was stored in a desiccator for approximately 1 h. LDI-TOF MSI experiments on grasshopper feces were performed in positive ion mode with 60 μm spatial resolution and within a mass range of *m/z* 200 to 1500.

#### Grass leaves/grasshoppers/excrements

All MSI experiments were performed on a UltrafleXtreme MALDI-TOF/TOF mass spectrometer (Bruker Daltonik GmbH) equipped with a smartbeam™ II laser. Each measurement was pre-calibrated externally using a commercial peptide calibration mixture (Bruker Daltonik GmbH) spotted on the same ITO-coated glass slide as the tissue at multiple positions. Subsequently, the measured datasets were visualized and partly analyzed using the software packages FlexImaging 4.0 (Bruker Daltonik GmbH) and SCiLS Lab 2015b (SCiLS GmbH, Bremen, Germany).

#### Water extractable soil organic matter (SOM)

Ten grams of homogenized fresh soil sample from 0 to 4 cm soil depth of the control and the grasshopper mesocosms was each weighted into a 45-mL centrifuge tube. Thirty milliliters of deionized water was added and horizontally shaken for 2 h at 150 rpm/min. After 5 min of centrifugation at 2800×*g*, the solutions were filtered under vacuum (0.45 μm pore size, cellulose acetate filter, Sartorius, Göttingen, Germany) and the resulting extracts were freeze-dried immediately. Subsequently, the extracts were re-suspended in 500 μL methanol and 1 μL was spotted on a MTP 384 target plate ground steel BC (Bruker Daltonik). The LDI-TOF MS experiments were performed as well on an UltrafleXtreme MALDI-TOF/TOF mass spectrometer in the positive ion mode within a mass range of *m/z* 150 to 2000. The resulting mass spectra are presented in Fig. 7.

#### Identification of secondary plant metabolites

All studied compartments, including grass, chewed grass (grass leaves containing grasshopper bites), grasshopper bodies, grasshopper excrements, topsoil (0 to 4 cm soil depth, root-free) of control and grasshopper mesocosms, were freeze-dried and carefully ground with a mixer mill (MM 200, Retsch, Haan, Germany) at room temperature and, subsequently, a 10 mg/mL methanol extract prepared. One microliter of the resulting extract was spotted on a MTP 384 target plate ground steel BC (Bruker Daltonik GmbH). All LDI-TOF MS and MS/MS experiments were performed on an UltrafleXtreme or Ultraflex III MALDI TOF/TOF mass spectrometer (Bruker Daltonik GmbH), operating in the positive or negative ion mode, employing argon at a pressure of 2.5 bar as collision gas in the MS/MS mode. The resulting MS/MS spectra were compared with the ones obtained from the reference standards to proof the identities of the secondary plant metabolites.

Additionally, 20 mg/mL methanol extracts was prepared from the studied compartments and analyzed by liquid-chromatography mass spectrometry (LC-MS) following a method described elsewhere [28]. In detail, the LC analysis was conducted on an Agilent 1100 Series LC system (Santa Clara, CA, USA) using a reversed phase column (EC 250/4.6 Nucleodur Sphinx, RP 5 μm, Macherey-Nagel, Düren, Germany). The solvent system comprised of 0.2% aqueous formic acid and acetonitrile using at flow rate of 1 mL/min at a temperature of 25 °C. The proportion of acetonitrile was increased from 10 to 50% in a linear gradient of 20 min followed by an increase to 75% in another 5 min. After the column was washed for 2 min with 100% acetonitrile, it was re-equilibrated to the initial eluent composition for 4 min prior to the next analysis. Mass spectra were recorded using an Esquire 6000 ESI-ion trap mass spectrometer (Bruker Daltonik GmbH). Typically, the secondary plant metabolites were analyzed in negative ion mode with a skimmer voltage of 60 V, a capillary exit voltage of −121 V, and a capillary voltage of 4000 V. Nitrogen was used as drying gas (11 mL/L, 330 °C) as well as nebulizer gas (pressure 35 psi). The secondary plant metabolites were identified by mass spectral data and chromatographic retention times in comparison to the reference standards.

## Results and discussion

### MALDI-TOF MSI analysis of a grass leaf

#### Method development

First, the MSI experiments were conducted with no matrix application in both, the positive and negative ion mode. However, the low number of detected signals led us to the conclusion that a matrix is required to increase the number of detectable ions. Hence, the common two matrices α-CHCA and DHB [9] were evaluated for the MALDI-TOF MSI analysis on grass leave surfaces. DHB (150 mg/mL in 50% methanol) sprayed with an air-brush device gave the highest number of detectable peaks in the positive ion mode and was therefore explored in more detail. It should be noted that the uneven leaf surface resulted in a mass variation of up to *m/z* ± 1, thus the shown ion images in Fig. 2 are generated using a mass window of up to *m/z* ± 1. To overcome this phenomenon, it is possible on the one hand to use a lock mass [29], or on the other hand to employ a newly developed recalibration software [30]; however, both approaches lie beyond the scope of this study. In order to prove that no interfering signals are present in the MALDI-TOF MSI dataset, two regions within the plant leaf were selected and total-ion-current (TIC) spectra constructed, as presented in the Electronic Supplementary Material (ESM) Fig. S3.

#### Plant response to insect herbivory

Polyphenols, which are produced by plants as a defense against aboveground herbivory [31], consist among others of simple phenols, phenolic acids, and flavonoids. All these compounds are readily soluble in methanol [32], hence a 50% methanol solution was used for the preparation of the matrix solution, so that we could assume to detect in particular polyphenols on the grass leaves with this method. We additionally analyzed the secondary metabolite standards (3-dehydroquinic acid, quinic acid, apigenin, luteolin, tricin, and rosmarinic acid) with DHB as matrix in the positive ion mode to prove their detection. The corresponding mass spectra can be found in the ESM Fig. S2.

As mentioned in the introduction, the question, which we would like to answer with the MALDI-TOF MSI experiments of grass leaves exposed to grasshoppers, is: Is it possible to visualize the response of herbivore feeding and mechanical damage of the grass leaves? Two kinds of reactions are likely: a rapidly propagating response throughout the plant or a response restricted to the wound site [33]. In our case, we observed the reduction of the concentration of certain compounds next to the wound, if a mechanical damage was performed shortly before the application of a matrix (see insert of Fig. 2a, b), and otherwise a nearly homogeneous distribution of defense compounds (see Fig. 2c–e). The mechanical damage (the area of the mechanical damage is denoted with a black square in Fig. 2b) caused a disruption of the cuticula of the leaf and the underlying secondary metabolites, such as quinic acid, apigenin, and luteolin, were presumably be oxidized leading to a decrease next to the wounded area. In two cases (Fig. 2b, f), a natural physiological gradient of the secondary metabolites is obtained. However, the secondary plant metabolites are already present in the leaves before the grasshopper attack, as measured by LDI-TOF MS analysis of ground grass leaves (see Table 2). A duration of 1 day between the feeding by grasshoppers and the subsequent analysis was selected in order to monitor the metabolic changes, which start after several hours and are still in progress after 1 day [34]. Since no increase of secondary plant metabolites next to the grasshopper bites are obtained, it is assumed that the time point of 1 day is maybe too short and the metabolic changes will only be visible to a later point in time [35].

#### Identification

**Fig. 3:**
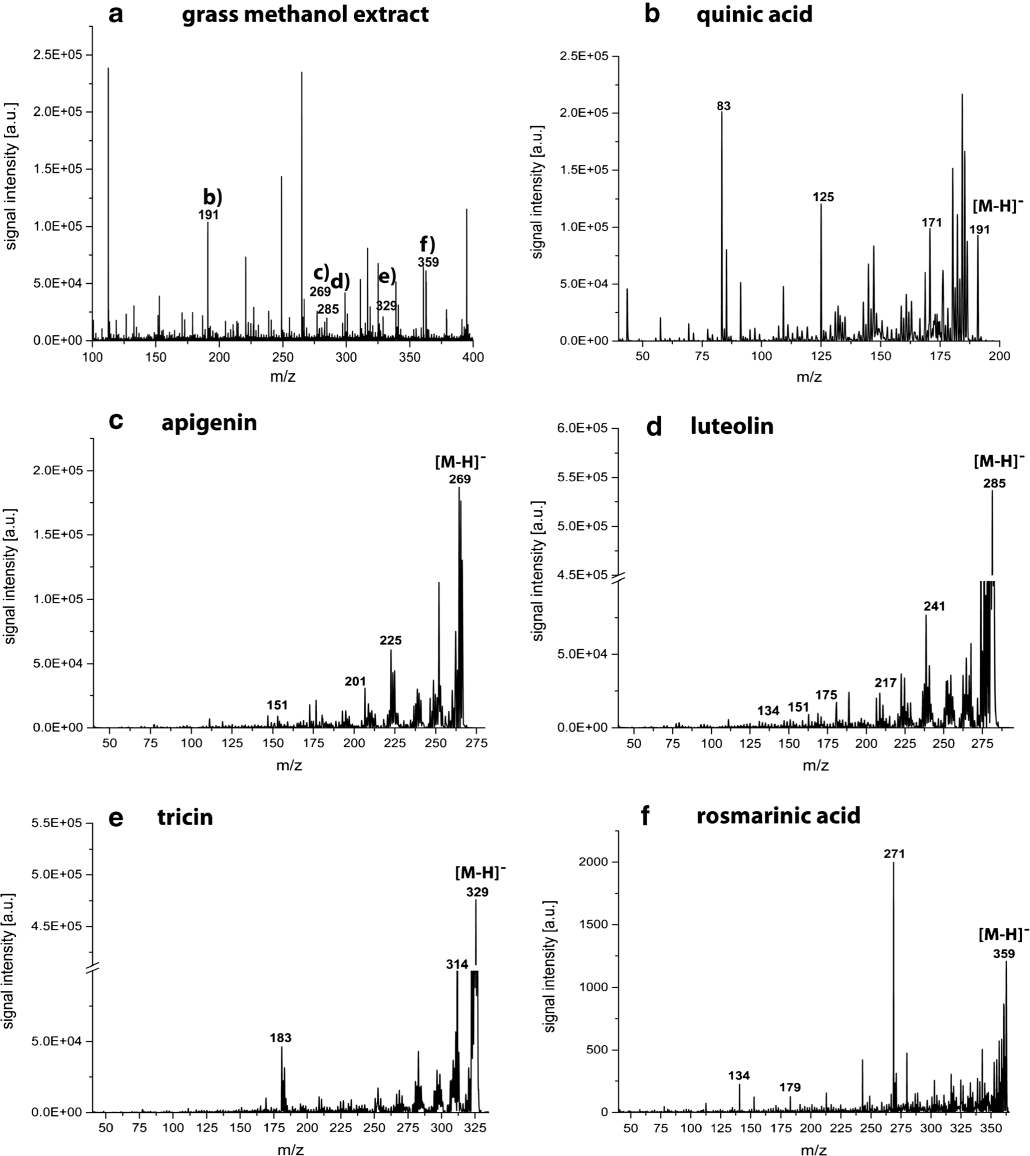
LDI-TOF MS and MS/MS spectra of a methanol extract from *D. glomerata* leaves in the negative ion mode for the identification of the secondary metabolites monitored in the ion images in Fig. 2. The MS/MS spectra of **b** quinic acid; **c** apigenin; **d** luteolin; **e** tricin; and **f** rosmarinic acid show the same diagnostic ions as for the reference substances summarized in Table 1

**Fig. 4:**
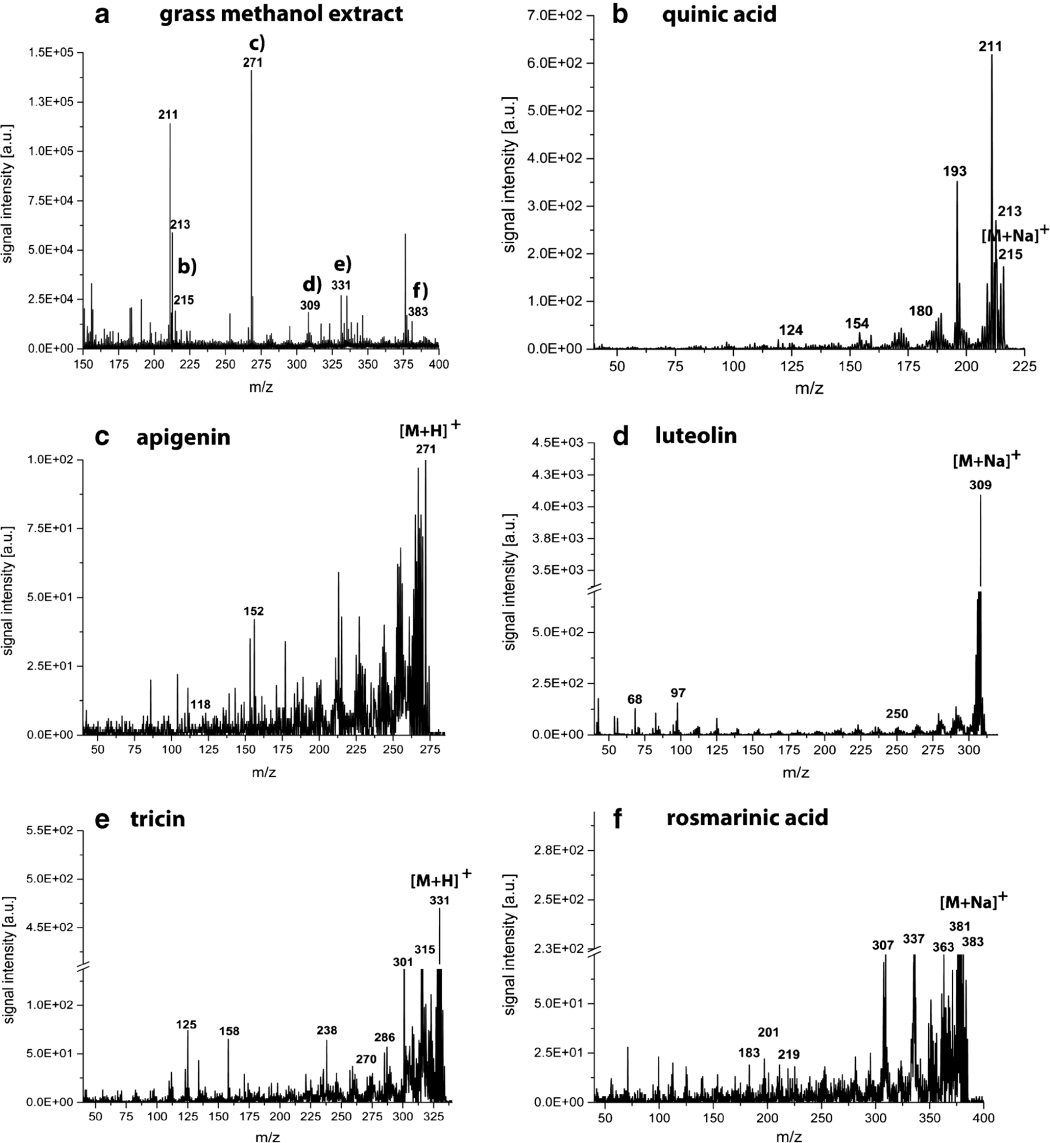
LDI-TOF MS and MS/MS spectra of a methanol extract from *D. glomerata* leaves in the positive ion mode for the identification of the secondary metabolites monitored in the ion images in Fig. 2. The ion images in Fig. 2 were as well recorded in the positive ion mode. The MS/MS spectra of **b** quinic acid; **c** apigenin; **d** luteolin; **e** tricin; and **f** rosmarinic acid show the same diagnostic ions as for the reference substances summarized in Table 1

The putative identification of the recorded secondary plant metabolites by on-tissue tandem MS analysis did not generate any useful data presumably due to the low sensitivity of this approach. Hence, putative secondary metabolite identities were assigned using MS/MS on the methanol extract of ground grass leaves and compared with reference standards. The MS and MS/MS spectra of the reference standards are presented in the ESM and the diagnostic ions are summarized in Table 1. The results generated from the methanol extract of grounded grass leaves are highlighted in Figs. 3 and 4, respectively. The identification of quinic acid, apigenin, luteolin, tricin, and rosmarinic acid was readily performed in both, the negative and positive ion mode. Both ion modes revealed diagnostic ions (see Table 1), which enabled the identification of the secondary plant metabolites, except of tricin, where no good mass accuracy could be observed. However, the glycosylated form of tricin was readily identified by LC-MS, as described later in this manuscript.

**Table 1:** Summary of diagnostic ions generated by LDI-TOF MS/MS analysis of reference standards. The corresponding spectra can be found in the ESM along with the structures of the secondary plant metabolites

Reference standard	Molecular formula	Negative ion mode		Positive ion mode	
		Detected ion (*m/z*)	Main fragment ions	Detected ion (*m/z*)	Main fragment ions
3-Dehydroquinic acid	C_7_H_10_O_6_	[M−H]^−^189	169, 125, 85	[M+Na]^+^213	211, 193, 180, 154, 124
Quinic acid	C_7_H_12_O_6_	[M−H]^−^ 191	171, 125, 83	[M+Na]^+^215	213, 211, 193, 180, 154, 124
Apigenin	C_15_H_10_O_5_	[M−H]^−^ 269	225, 201, 151	[M+H]^+^271	271, 152, 118
Luteolin	C_15_H_10_O_6_	[M−H]^−^ 285	241, 217, 199, 175, 151, 134	[M+Na]^+^309	279, 250, 97, 68
Tricin^a^	C_17_H_14_O_7_	[M−H]^−^ 329	314, 298, 284, 150	[M+H]^+^331	315, 301, 286, 270, 239, 178, 153
Rosmarinic acid	C_18_H_16_O_8_	[M−H]^−^ 359	271, 197, 179, 161, 134	[M+Na]^+^383	381, 363, 337, 307, 219, 201, 183

^a^Tricin is an in-source decay (ISD) product of the reference standard tricin-5-glycoside. Due to deviation in the mass accuracy, it is not considered as tentatively identified by LDI-TOF MS/MS

### LDI-TOF MSI of a grasshopper

#### Method development

Preliminary data were obtained by testing a variety of different matrices typically employed for the MALDI-TOF MSI analysis of metabolites [6], such as DHB, dithranol, and 1,5-DAN in the positive and negative reflector mode, respectively. However, only the simplest sample preparation by leaving out the matrix application enabled us to use a spatial resolution of 50 μm, additionally, this technique prevented the delocalization of the studied compounds, which was observed, in particular when DHB was sprayed with an air-brush device on top of the grasshopper longitudinal section. The grasshopper longitudinal section was not further investigated in the current study, since it was impossible to track the secondary plant metabolites in the sections. Hence, further analysis was conducted on the extracted gastrointestinal tract.

#### Gastrointestinal tract

**Fig. 5:**
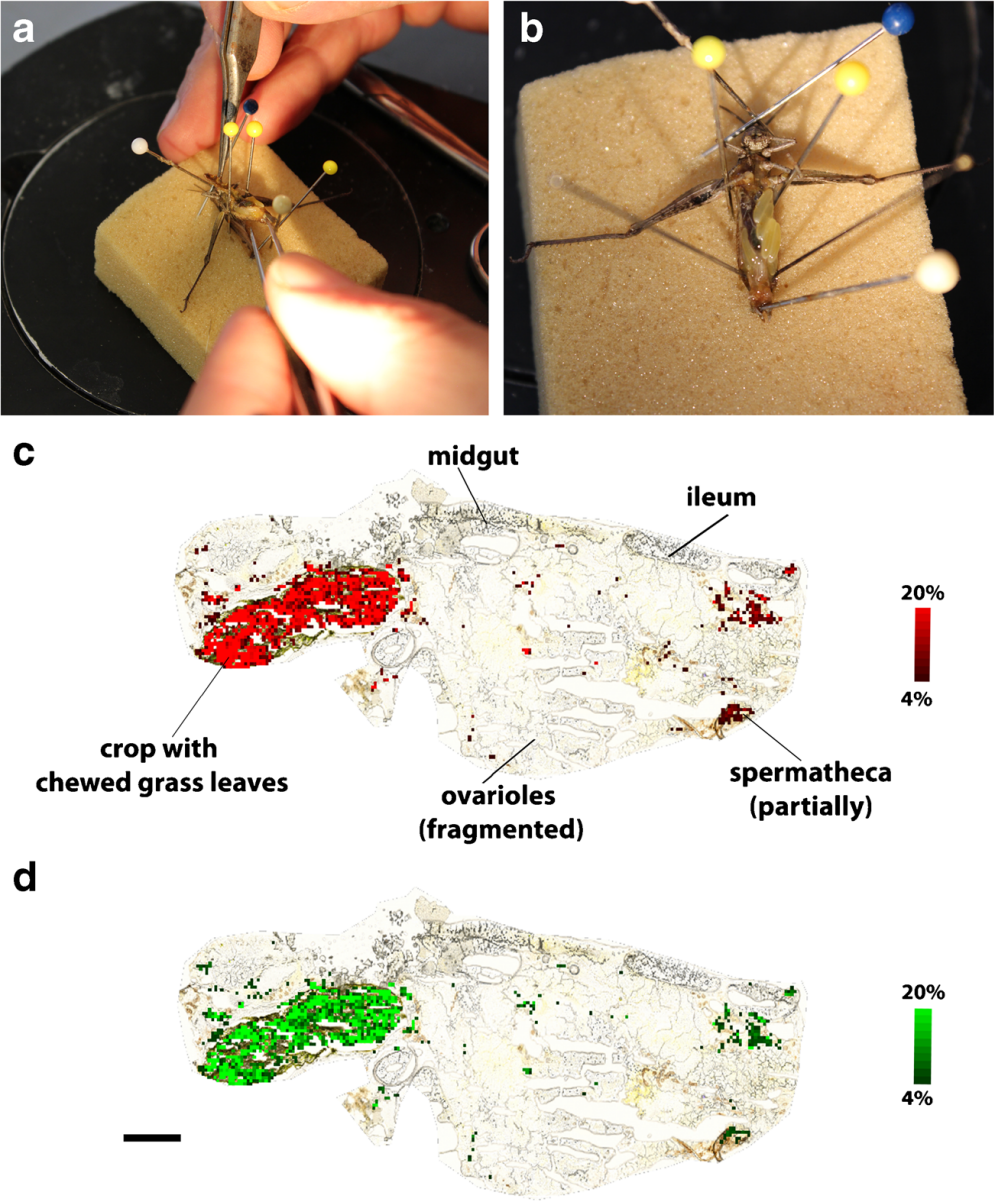
LDI-TOF MSI analysis of the gastrointestinal tract of a female *C. dorsatus*. The photographs show **a** the fixation of the partly frozen animal, and **b** the halfway extracted gastrointestinal tract. Ion images from the LDI-TOF MSI analysis present the distribution of **c** dehydroquinic acid (*m/z* 213, [M+Na]^+^, *red color*) and **d** quinic acid (*m/z* 215, [M+Na]^+^, *green color*), overlaid on the optical image taken before MSI Note: The assignments of the secondary plant metabolites are tentative based on MS and MS/MS experiments of methanol extracts of *C. dorsatus*, as summarized in Table 2 and compared with reference standards, as presented in the ESM. The ion images are normalized using the TIC. *Scale bar* 1000 μm (**d**)

The gastrointestinal tract was extracted from a grasshopper body that was emerged for only 1 s in liquid nitrogen, as shown in Fig. 5a, b to study the route of the eaten grass leaves and with it the studied polyol and phenolic compounds in the grasshopper. Since the complete gastrointestinal tract was not embedded in an appropriate embedding medium, some organs are squeezed and displaced, as shown in Fig. 5c, d. Therefore, only the organs, which could be assigned confidently, are marked in Fig. 5c. The ion images of dehydroquinic acid (*m/z* 213, [M+Na]^+^, red color) and quinic acid (*m/z* 215, [M+Na]^+^, green color) are overlaid on the optical image as presented in panels c and d of Fig. 5, respectively. To show the signal intensities of the MSI-related signals and the background, two regions within the gastrointestinal tract were selected and TIC spectra constructed, as presented in the ESM Fig. S11. Except for the crop, only noise could be recorded in the other organs of the grasshopper. Dehydroquinic acid is an in-source-decay (ISD) product of quinic acid, since it can be detected measuring the reference standard quinic acid. To confirm that the reference standard quinic acid is not degraded, resulting in the formation of dehydroquinic acid, a proton nuclear magnetic resonance (^1^H NMR) spectrum was recorded depicting all required protons in the molecule. The corresponding ^1^H NMR spectrum can be found in the ESM Fig. S10. Both secondary plant metabolites, dehydroquinic acid and quinic acid, can only be detected in the crop of the grasshopper, which is filled with chewed grass leaves.

The four remaining phenolic compounds, apigenin, luteolin, tricin, and rosmarinic acid, could not be detected in the crop, since they are presumably already digested in the crop. The identification of dehydroquinic acid and quinic acid was performed by MS/MS analysis on methanol extracts of ground grasshoppers and compared to the corresponding MS/MS spectra generated from the reference standards (see ESM and Table 1). The MS and MS/MS experiments on methanol extracts of the grasshoppers were performed in both, the negative and positive ion mode, as summarized in Table 2. In the negative ion mode, apigenin and luteolin and no dehydroquinic acid and quinic acid could be detected. However, in the positive ion mode dehydroquinic acid and luteolin could be identified. The results from the negative and positive ion mode do not completely agree, highlighting the importance of the selected ion mode. The observed difference might originate from the diverse sensitivity of the ions in the corresponding ion mode.

**Table 2:** Summary of LDI-TOF MS and MS/MS data from methanol extracts of studied compartments. (x, present; −, absent)

Tentative assignment	Molecular formula	Negative ion mode							Positive ion mode						
		Detected ion (*m/z*)	Grass	Chewed grass	Grasshoppers	Excrements	Soil (control)	Soil with grasshoppers	Detected ion (*m/z*)	Grass	Chewed grass	Grasshoppers	Excrements	Soil (control)	Soil with grasshoppers
Dehydroquinic acid	C_7_H_10_O_6_	[M−H]^−^189	−	−	−	−	−	−	[M+Na]^+^213	x	x	x	x	x	x
Quinic acid	C_7_H_12_O_6_	[M−H]^−^191	x	x	−	−	−	−	[M+Na]^+^215	x	x	x	x	−	−
Apigenin	C_15_H_10_O_5_	[M−H]^−^269	x	x	x	x	−	−	[M+H]^+^271	x	x	−	−	−	−
Luteolin	C_15_H_10_O_6_	[M−H]^−^285	x	x	x	x	−	−	[M+Na]^+^309	x	x	x	x	x	x
Tricin	C_17_H_14_O_7_	[M−H]^−^329	x	x	−	−	−	−	[M+H]^+^331	x	x	−	−	−	−
Rosmarinic acid	C_18_H_16_O_8_	[M−H]^−^359	x	x	−	−	−	−	[M+Na]^+^383	x	x	−	−	−	−

The post-digestive effects of polyphenols usually occur in the midgut of insects through oxidative mechanisms, resulting in the formation of superoxide radicals and other reactive oxygen species [36]. The question, which is coming up is, do the considered metabolites really digest and, with this, provide the required energy for the grasshopper, or do they stay undigested and hence, can be found in the excrements. To answer this question, the excrements of the grasshoppers were analyzed by LDI-TOF MSI.

### LDI-TOF MSI of grasshopper excrements

#### Method development

The considered secondary plant metabolites (quinic acid, apigenin, luteolin, tricin, and rosmarinic acid) were detectable in the grass leaf and partially as well in the grasshopper employing the positive ion mode, hence this mode was furthermore used for the analysis of excrements. The matrix DHB was sprayed with an air-brush device on top of the excrements or as alternative no matrix was used. The LDI-TOF MSI analysis will be considered here in detail, since the number of detectable signals was higher than the ones obtained for the MALDI-TOF MSI analysis using DHB as matrix.

#### Undigested plant metabolites in excrements

**Fig. 6:**
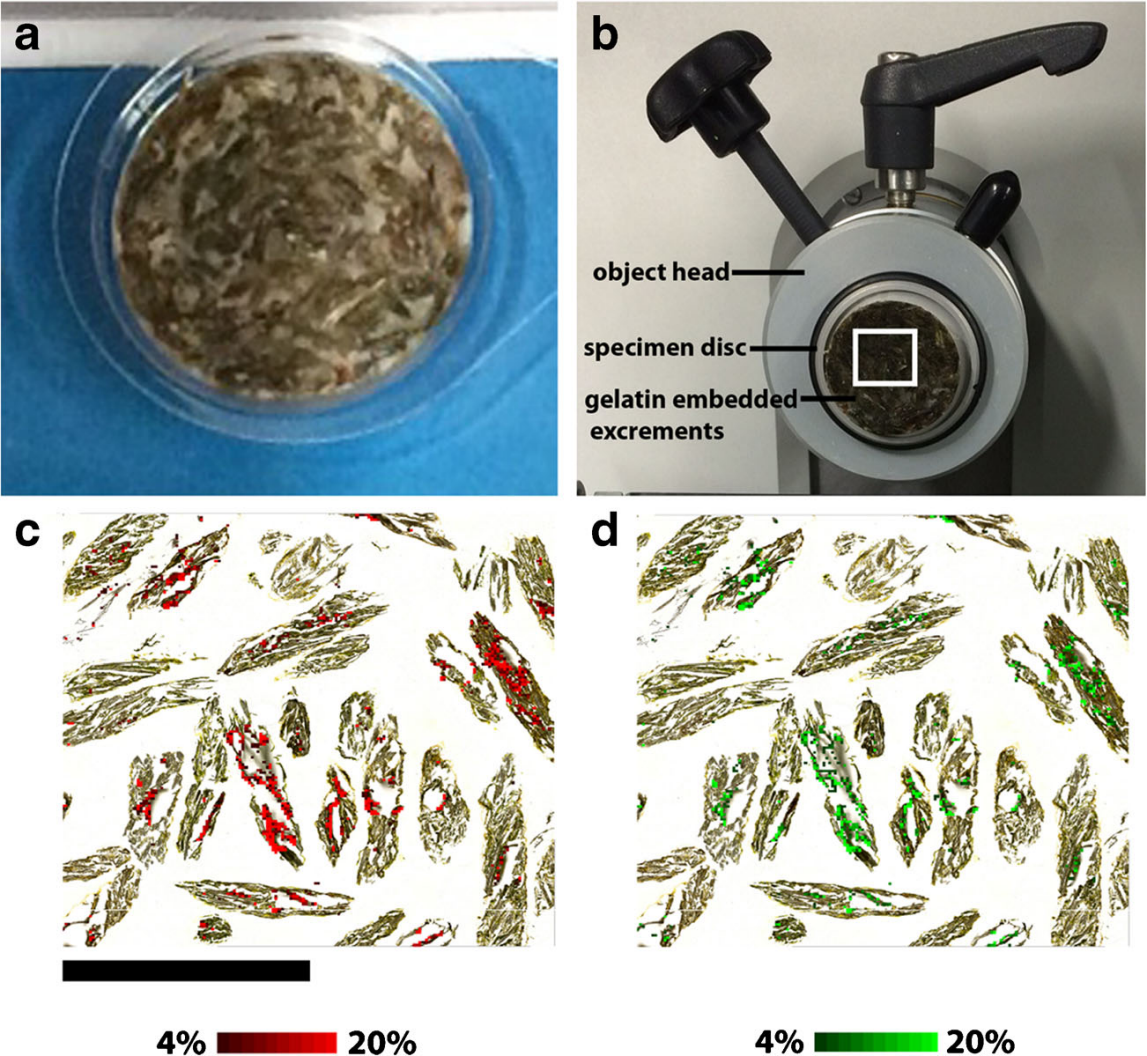
LDI-TOF MSI analysis of excrements of *C. dorsatus*. The photographs show **a** excrements embedded in 10% gelatin in a disposable specimen mold; **b** view into the cryostat where the embedded excrements are attached with BPS onto the specimen disc, which is mounted on the object head. The ion images generated by LDI-TOF MS of dehydroquinic acid (*m/z* 213, [M+Na]^+^, *red color*) and quinic acid (*m/z* 215, [M+Na]^+^, *green color*) are overlaid on the optical image in picture **c** and **d**, respectively Note: The assignments of the secondary plant metabolites are tentative based on MS and MS/MS experiments of methanol extracts of the excrements, as summarized in Table 2 and compared with reference standards, as presented in the ESM. The ion images are normalized using the TIC. *Scale bar* 5 mm (**c**)

The collected excrements were embedded in 10% gelatin and placed in a disposable specimen mold, as shown in Fig. 6a. Afterwards, the frozen and embedded excrements were attached to the specimen disc and mounted onto the object head in a cryostat, as presented in Fig. 6b. The subsequent LDI-TOF MSI analysis of a square (1 cm × 1 cm) of a 12-μm-thick section revealed only the presence of dehydroquinic acid and quinic acid in the excrements. The signal intensities of the MSI-related signals and the background from two selected regions within the excrements section is presented in the ESM in Fig. S12. In region 1 only, the signals of dehydroquinic acid and quinic acid are visible in the constructed TIC spectrum, whereas in region 2 strong signals between *m*/*z* 400 and 600 are observed and no signals of the two considered secondary metabolites. This proves the partially presence of dehydroquinic acid and quinic acid in the analyzed excrement section, as shown in panel c and d of Fig. 6, respectively. The identification of both metabolites was as well confirmed by MS/MS (see Table 2), as described previously, and compared with the data obtained from the reference standards (see ESM and Table 1). The analysis of the methanol extracts of the excrements revealed in the negative ion mode the presence of apigenin and luteolin; and in the positive ion mode the presence of dehydroquinic acid and luteolin. It is surprising that luteolin is detected in both ion modes in the methanol extracts, although it is not detected in the MSI data, which are recorded in the positive ion mode. Maybe the concentration of luteolin is not sufficient in the cut of the excrements on the micrometer scale, and hence is not detectable. The preparation of the methanol extracts with 10 mg/mL is fairly high and can be considered as an enrichment step. To validate the occurrence of luteolin in a single excrement slice, we scratched a single excrement slice from the ITO-coated glass slide and subsequently prepared a methanol extract for removing any potential matrix effects. Indeed, we could detect luteolin by LDI-TOF MS in the methanol extract. This phenomenon reflects the disadvantage of the present MSI technique compared to solution-based MS, the sensitivity in solution-based MS is commonly higher by means of detection, e.g., more compound signals.

The obtained ion images of dehydroquinic acid and quinic acid overlaid on the optical image acquired prior MSI can be viewed in panels c and d of Fig. 6, respectively. It can be concluded from Fig. 6 that only the cyclic polyol compounds remain undigested by the grasshoppers and are released while the other four investigated phenolic compounds are maybe used to gain energy or to form reactive oxygen species.

### LDI-TOF MS of soil organic matter

#### Presence of secondary plant metabolites in water extractable SOM

**Fig. 7:**
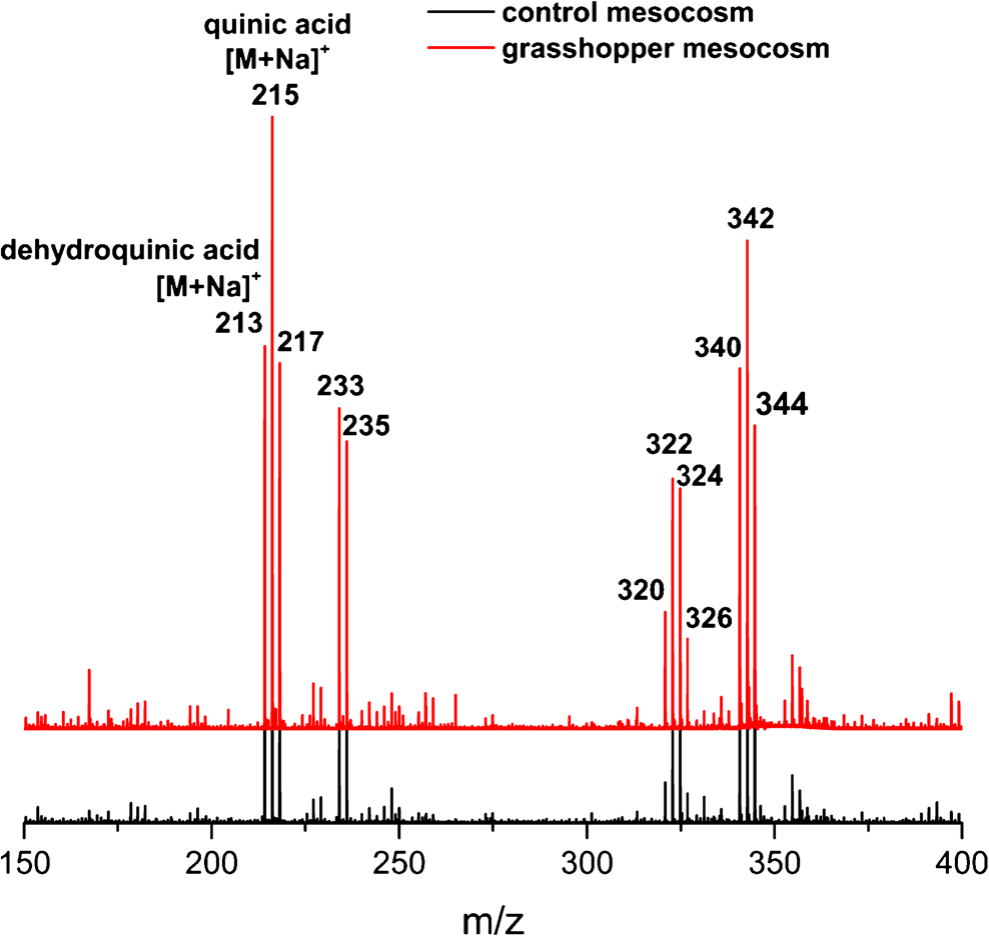
LDI-TOF MS analysis of water extractable SOM in both, control (*black line color*) and grasshopper (*red line color*) mesocosms in the positive ion mode. The secondary plant metabolites dehydroquinic acid (*m/z* 213, [M+Na]^+^) and quinic acid (*m/z* 215, [M+Na]^+^) are readily visible in both mesocosms and were putative assigned by MS/MS analysis

The answer, which is still missing is, if these cyclic polyols finally enter the soil via rapid leaching of excrements or not. As shown in Fig. 7, dehydroquinic acid with an *m/z* value of 213 [M+Na]^+^ and quinic acid with an *m/z* value of 215 [M+Na]^+^ are readily visible as compounds of water extractable SOM in both control and grasshopper mesocosms. The assignment of the signals was performed by MS/MS analysis and diagnostic ions from the spectra correspond to the ones obtained from the reference standards (summarized in Table 1). Hence, dehydroquinic acid and quinic acid can not only enter the soil as leachates from excrements but also from the grass leaves itself as decomposition products [37].

#### Presence of secondary plant metabolites in methanol extractable SOM

**Fig. 8:**
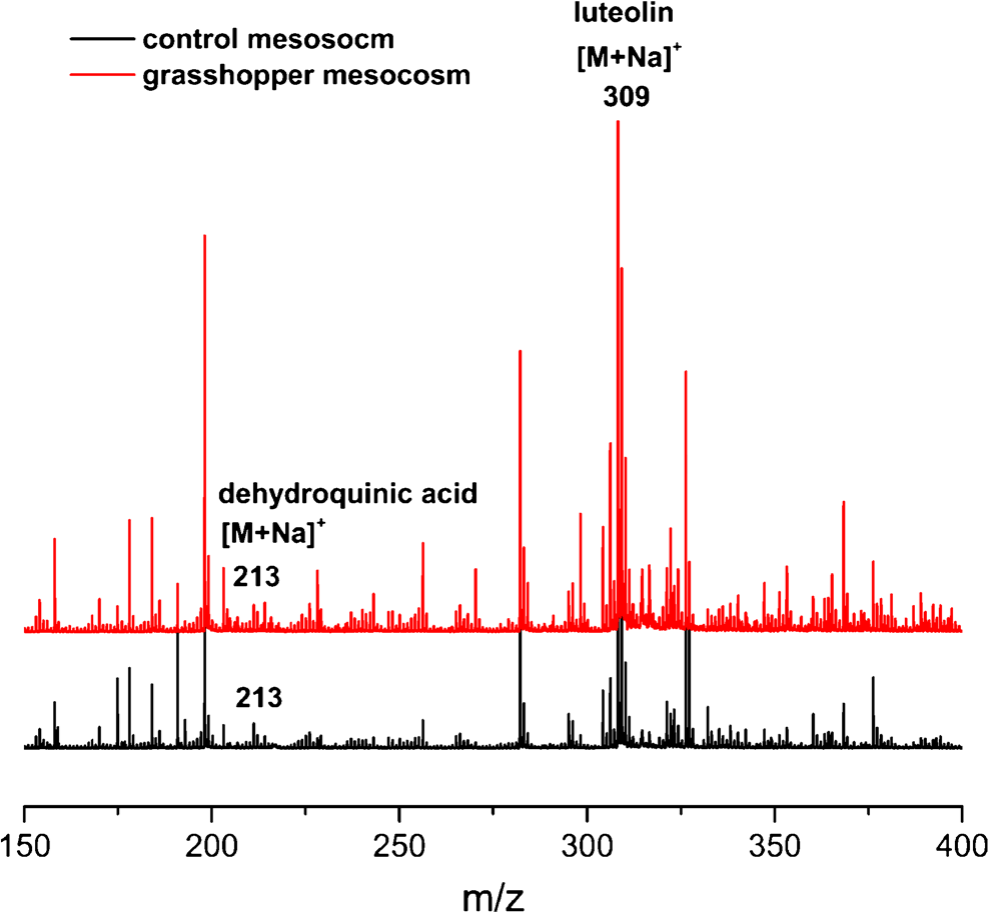
LDI-TOF MS analysis of methanol extractable SOM in both, control (*black line color*) and grasshopper (*red line color*) mesocosms in the positive ion mode. The secondary plant metabolites dehydroquinic acid (*m/z* 213, [M+Na]^+^) and luteolin (*m/z* 309, [M+Na]^+^) are readily visible in both mesocosms and were putative assigned by MS/MS analysis

The last answer, which needs to be addressed is, if the cyclic polyol compounds detected in the organic substances of the soil solution can also be found in the more stable SOM matter pool of both soil samples of control and grasshopper mesocosms. In order to approach this last step, a methanol extract of the topsoil (0–4 cm soil depth) sampled from the mesocosms was prepared and the resulting LDI-TOF MS spectra are presented in Fig. 8. The recorded spectra in Fig. 8 are more complex, than the ones recorded in Fig. 7, since Fig. 8 considers the bulk soil samples and not only the water extractable part. Dehydroquinic acid with an *m/z* value of 213 [M+Na]^+^, and luteolin with an *m/z* value of 309 [M+Na]^+^, are readily visible in both topsoil materials, whereas the three remaining secondary plant metabolites (apigenin, tricin, and rosmarinic acid) cannot be detected, since they have likely been converted in the grasshoppers body or formed insoluble complexes with soil proteins [20]. The MS/MS experiments from dehydroquinic acid and luteolin confirm the presence of both secondary plant metabolites. In comparison with LDI-TOF MS of water extractable SOM, quinic acid is not present in the bulk soil, since it is presumably completely converted to dehydroquinic acid, as an ISD product. However, this secondary plant metabolite is only detectable in the positive ion mode. In summary, dehydroquinic acid can also be found in the bulk soil samples, independently from herbivory (see Table 2).

### LC-MS analysis of methanol extracts of the studied compartments

From the studied compartments, including grass leaves, chewed grass leaves, grasshoppers, excrements, soil of control and grasshopper mesocosms, methanol extracts of the grounded material were prepared and subsequently analyzed by LC-MS. The obtained results are summarized in Table 3. Besides quinic acid, derivatives of the acid are detected in LC-MS, such as caffeoyl quinate and ferroyl quinate. Both compounds are not detectable with LDI-TOF MS, however the signal of quinic acid comes partly from them. To proof this hypothesis, the derivative cryptochlorgenic acid was purchased and analyzed by LDI-TOF MS. (Since the derivatives caffeoyl quinate and ferroyl quinate were unavailable, cryptochlorgenic acid was used instead.) In the recorded mass spectrum, a signal originating from quinic acid was obtained, which was formed as an ISD product from cryptochlorgenic acid. The same case was obtained for the polyphenols apigenin, luteolin, and tricin. Here, only the glycosylated forms can be detected with LC-MS. Since only the 5-glycoside forms were purchasable, they were used to show that these derivatives show in LDI-TOF MS a signal for the pure polyphenol without the sugar moiety. Hence, it can be concluded that apigenin, luteolin, and tricin are unsuitable biomarkers to trace DOM, since they are formed as ISD products from the glycosylated structures.

**Table 3:** Summary of LC-MS data from methanol extracts of studied compartments. (x, present; −, absent)

Tentative assignment	Molecular formula	R_t_ (min)	Reference	Detected ion (*m/z*)	Grass	Chewed grass	Grasshoppers	Excrements	Soil (control)	Soil with grasshoppers
Dehydroquinic acid	C_7_H_10_O_6_			[M−H]^−^ 189	**–**	**–**	**–**	**–**	**–**	**–**
Quinic acid	C_7_H_12_O_6_	2.8	[32]	[M−H]^−^ 191	x	x	–	–	–	–
Caffeoyl quinate	C_16_H_18_O_9_	3 isomers at 7.3, 9.1, 11.3	[38]	[M−H]^−^ 353	x	x	–	–	–	–
Ferroyl quinate	C_17_H_20_O_9_	9.4	[38]	[M−H]^−^ 367	x	x	–	–	–	–
Apigenin	C_15_H_10_O_5_		[32]	[M+H]^+^ 269	–	–	–	–	–	–
Apigenin-hexoside-pentoside	C_26_H_28_O_15_	10.5	[38]	[M−H]^−^ 563	x	x	x	x	–	–
Luteolin	C_15_H_10_O_6_		[32]	[M−H]^−^ 285	–	–	–	–	–	–
Luteolin-hexoside-pentoside	C_26_H_28_O_15_	9.5	[38]	[M−H]^−^ 579	x	x	x	x	–	–
Tricin	C_17_H_14_O_7_		[38]	[M+H]^−^ 331	–	–	–	–	–	–
Tricin-hexoside	C_23_H_24_O^12^	13.0	[39]	[M–H]- 491	x	x	–	–	–	–
Rosmarinic acid	C_18_H_16_O_8_	15.7	[38]	[M–H]^−^ 359	x	x	–	–	–	–

The LC-MS data highlight the presence of the following secondary metabolites in both compartments grass and chewed grass: quinic acid, caffeoyl quinate, ferroyl quinate, tricin-hexoside, rosmarinic acid, apigenin-hexoside-pentoside, and luteolin-hexoside-pentoside. Using the LC-MS data to validate the MSI data, as summarized in Table 4, it can be concluded that the MSI data show the same-targeted secondary compounds present in the leaf, as the LC-MS data, taking into account that ISD products of quinic acid, tricin, apigenin, and luteolin were formed by LDI-TOF MSI. Furthermore, apigenin-hexoside-pentoside and luteolin-hexoside-pentoside could be detected up to the grasshoppers’ excrements by LC-MS analysis. Hence, one would expect to detect apigenin and luteolin in the grasshopper crop and excrements by LDI-TOF MSI. However, this is not the case. Only quinic acid and its ISD product dehydroquinic acid could be determined by MSI. One suitable explanation is the presumably higher sensitivity of the glycosylated forms of apigenin and luteolin by LC-MS analysis compared to the non-glycosylated forms by the imaging technique LDI-TOF MSI. By preparing a MeOH extract from a scrapped off excrement slice, one could detect luteolin by LDI-TOF MS, underlining the sensitivity differences between MSI and solution-based MS, as described early in this manuscript.

**Table 4:** Summary of MSI data. (x, present; −, absent)

Tentative assignment	Molecular formula	Detected ion (*m/z*)	Chewed grass	Grasshopper	Excrements
Dehydroquinic acid	C_7_H_10_O_6_	[M+Na]^+^213	−	x	x
Quinic acid	C_7_H_12_O_6_	[M+Na]^+^215	x	x	x
Apigenin	C_15_H_10_O_5_	[M+H]^+^271	x	−	−
Luteolin	C_15_H_10_O_6_	[M+Na]^+^309	x	−	−
Tricin	C_17_H_14_O_7_	[M+H]^+^331	x	−	−
Rosmarinic acid	C_18_H_16_O_8_	[M+Na]^+^383	x	−	−

Surprisingly are the strong signals originating from quinic acid and its ISD product dehydroquinic acid by LDI-TOF MSI in the positive ion mode. The easy ionization of this secondary plant metabolite by LDI in the positive ion mode might be one reason of its appearance throughout all compartments. Indeed, in the negative ion mode, dehydroquinic acid is not measurable in any compartment by LDI-TOF MS (see Table 2); moreover, quinic acid is only detectable in the control and chewed grass highlighting the differential ionization efficiency of the two considered secondary plant metabolites in the diverse ion modes.

## Conclusion

In summary, the present study demonstrated the implementation of the analytical techniques LDI-TOF MSI and MALDI-TOF MSI to follow the fate and transport of various secondary plant metabolites, in particular two cyclic polyols and four polyphenols. The secondary plant metabolites were traced from the grass leaves of *D. glomerata*, which were taken up by the grasshopper *C. dorsatus*, or deposited as excrements, finally ending up in the soil extracts and bulk soil. Table 4 presents a short summary of the MSI data. Only the cyclic polyols, dehydroquinic acid, which is formed from quinic acid as an ISD product, and quinic acid itself followed this route, the four polyphenols, apigenin, luteolin, tricin, and rosmarinic acid, were not deposited as excrements. This can have several reasons: The specific polyphenols (1) can be converted in the grasshoppers’ crop, (2) can be digested in the grasshoppers’ midgut, (3) can form reactive superoxide species in the grasshoppers’ midgut [36], or (4) can build up insoluble protein-complexes in the soil [40]. The identification of the considered secondary plant metabolites was performed on methanol extracts of the various compartments and compared with the MS/MS data generated from the corresponding reference standards, since the on-tissue MS/MS analysis was not sensitive enough. Additional LC-MS data were acquired from methanol extracts from the compartments and apigenin-hexoside-pentoside and luteolin-hexoside-pentoside could be traced up to the grasshoppers’ excrements in contrast to the MSI results. By tacking the LC-MS results to verify the MSI results, it became obvious that the MSI technique, although providing spatial information, which cannot be gained by LC-MS, suffered from sensitivity issues. Hence, the combination of MSI and LC-MS is the way to approach environmental questions, as described here. In conclusion, no ideal secondary plant metabolite could be found to function as a biomarker for tracing the source of DOM in soil upon insect herbivory. Even though, quinic acid, which owns a potential allelophatic role by modulating plant community composition [37], could be traced in all compartments by MSI, the LC-MS data did not proof this behavior. The comparison of both MS techniques, MSI and LC-MS, mirrors the complexity of the studied system.

## Electronic supplementary material

ESM 1 (PDF 1754 kb)
