# Supplementary material for: Tracing the fate and transport of secondary plant metabolites in a laboratory mesocosm experiment by employing mass spectrometric imaging
Source: Anal Bioanal Chem. 2017 Mar 29;409(15):3807–20. doi: 10.1007/s00216-017-0325-7 (PMC5427159; doi:10.1007/s00216-017-0325-7)
Supplement: Supplementary file 1 — (PDF 1754 kb) [file 216_2017_325_MOESM1_ESM.pdf]

Analytical and Bioanalytical Chemistry

Electronic Supplementary Material

Tracing the fate and transport of secondary plant metabolites in a laboratory mesocosm experiment by employing mass spectrometric imaging

Anna C. Crecelius, Beate Michalzik, Karin Potthast, Stefanie Meyer, Ulrich S. Schubert

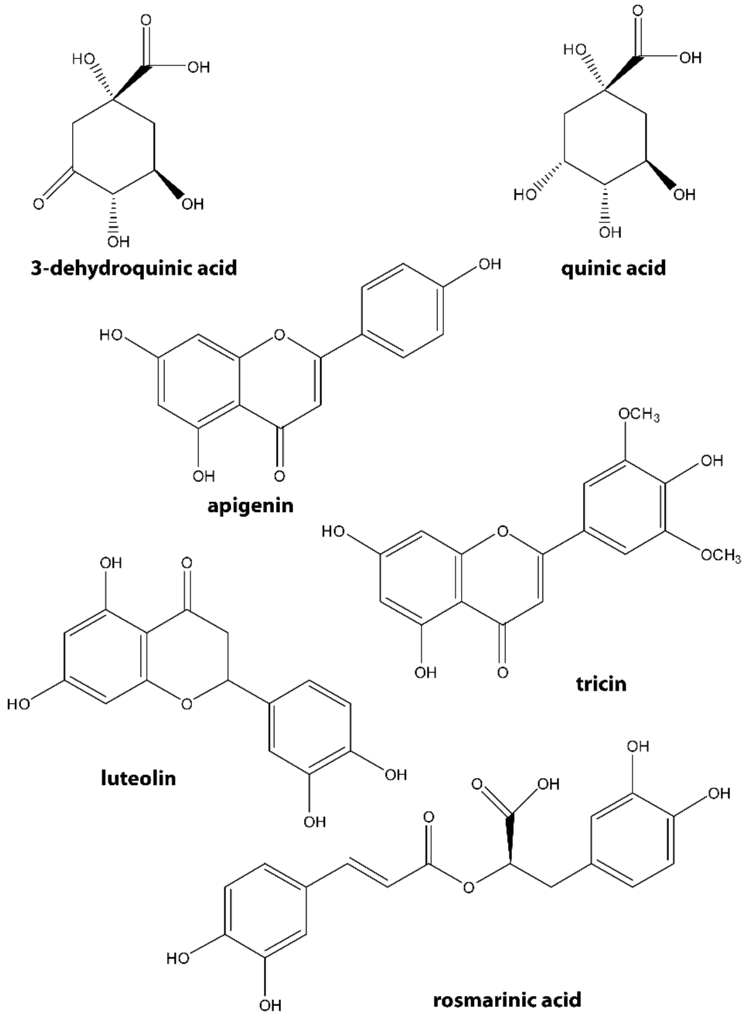

Fig. S1 Structures of secondary metabolites identified in *Dactylis glomerata* leaves

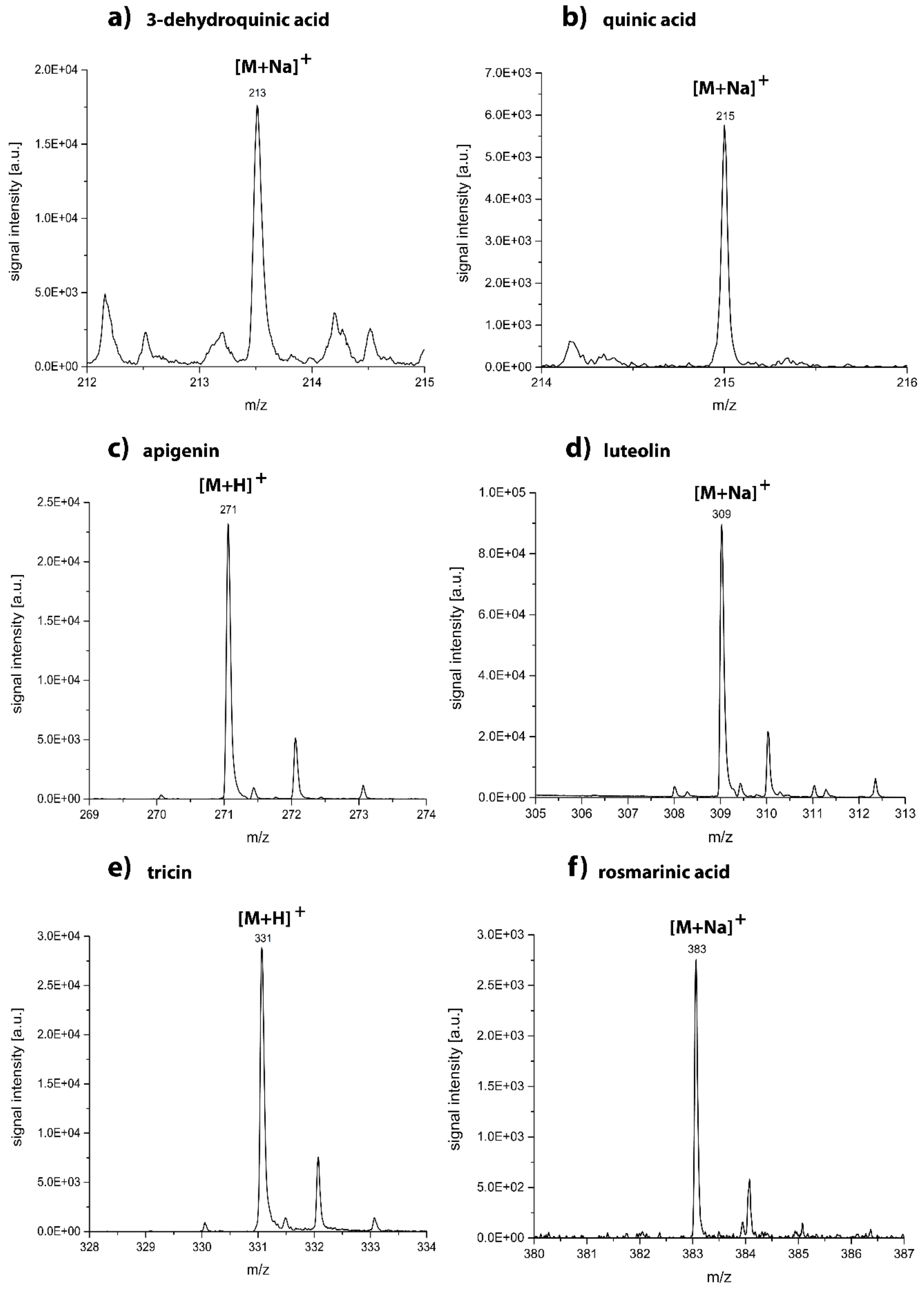

Fig. S2 MALDI-TOF MS spectra of the reference standards **a)** 3-dehydroquinic acid, **b)** quinic acid, **c)** apigenin, **d)** luteolin, **e)** tricin (generated as in-source decay product from the reference standard tricin-5-glycoside), and **f)** rosmarinic acid in positive ion mode using DHB as matrix

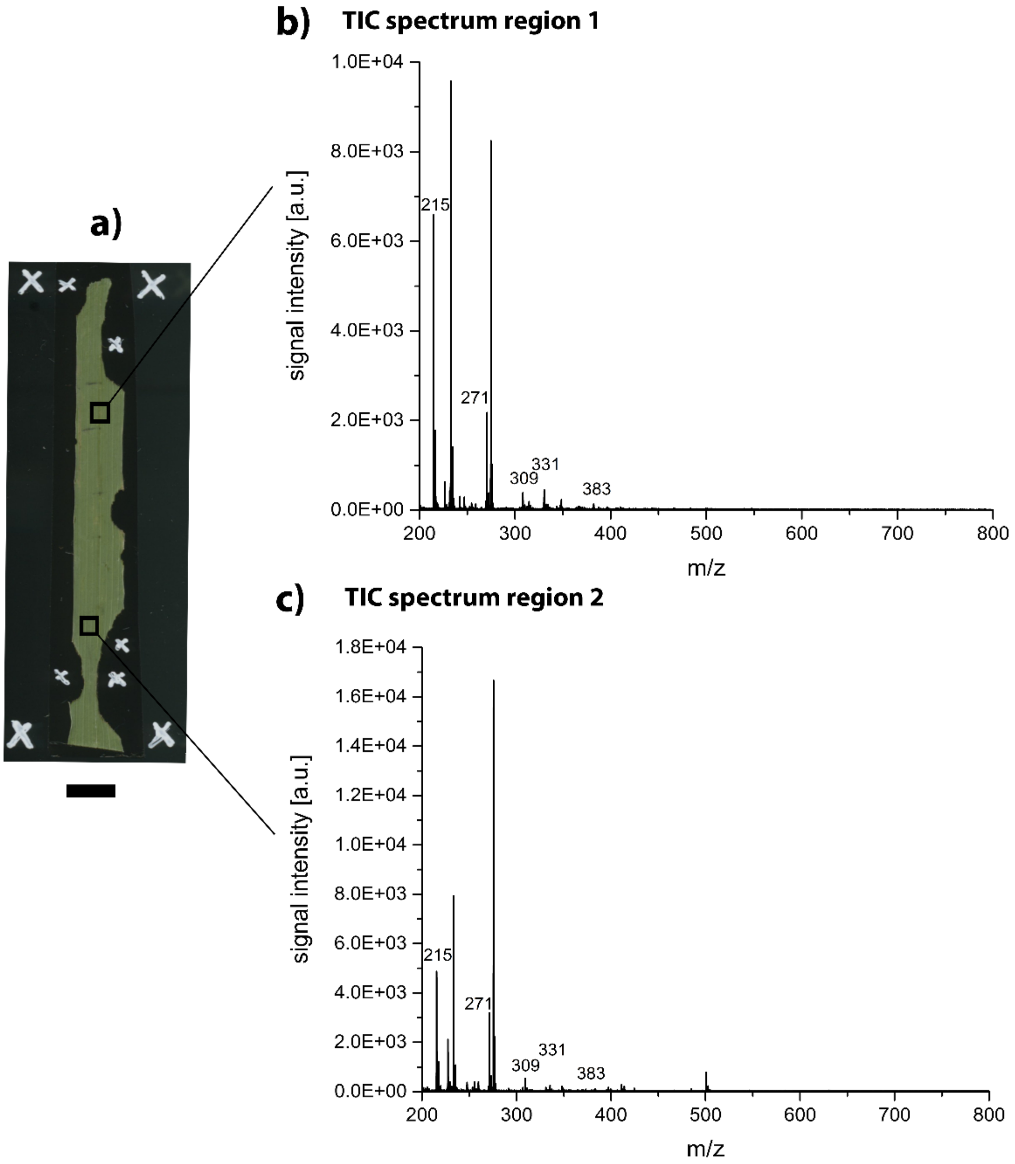

Fig. S3 MALDI-TOF MSI dataset of a *Dactylis glomerata* leaf. **a)** Optical image taken before the matrix application, indicating two areas, from which TIC spectra were constructed, **b)** TIC spectrum from region 1, **c)** TIC spectrum from region 2. Scale bar 5 mm **a)**

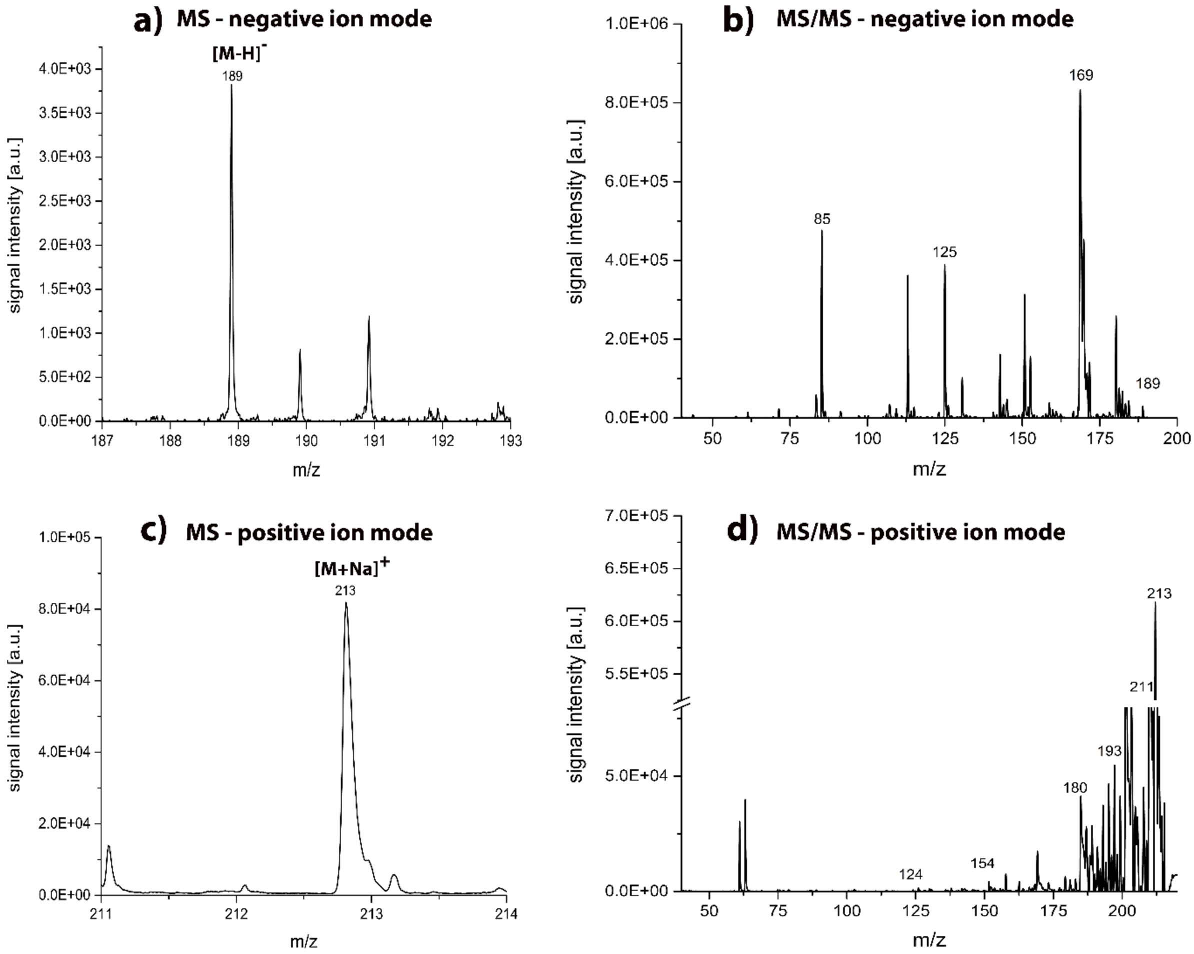

Fig. S4 LDI-TOF MS and MS/MS spectra of the reference standard 3-dehydroquinic acid in the negative and positive ion mode, respectively

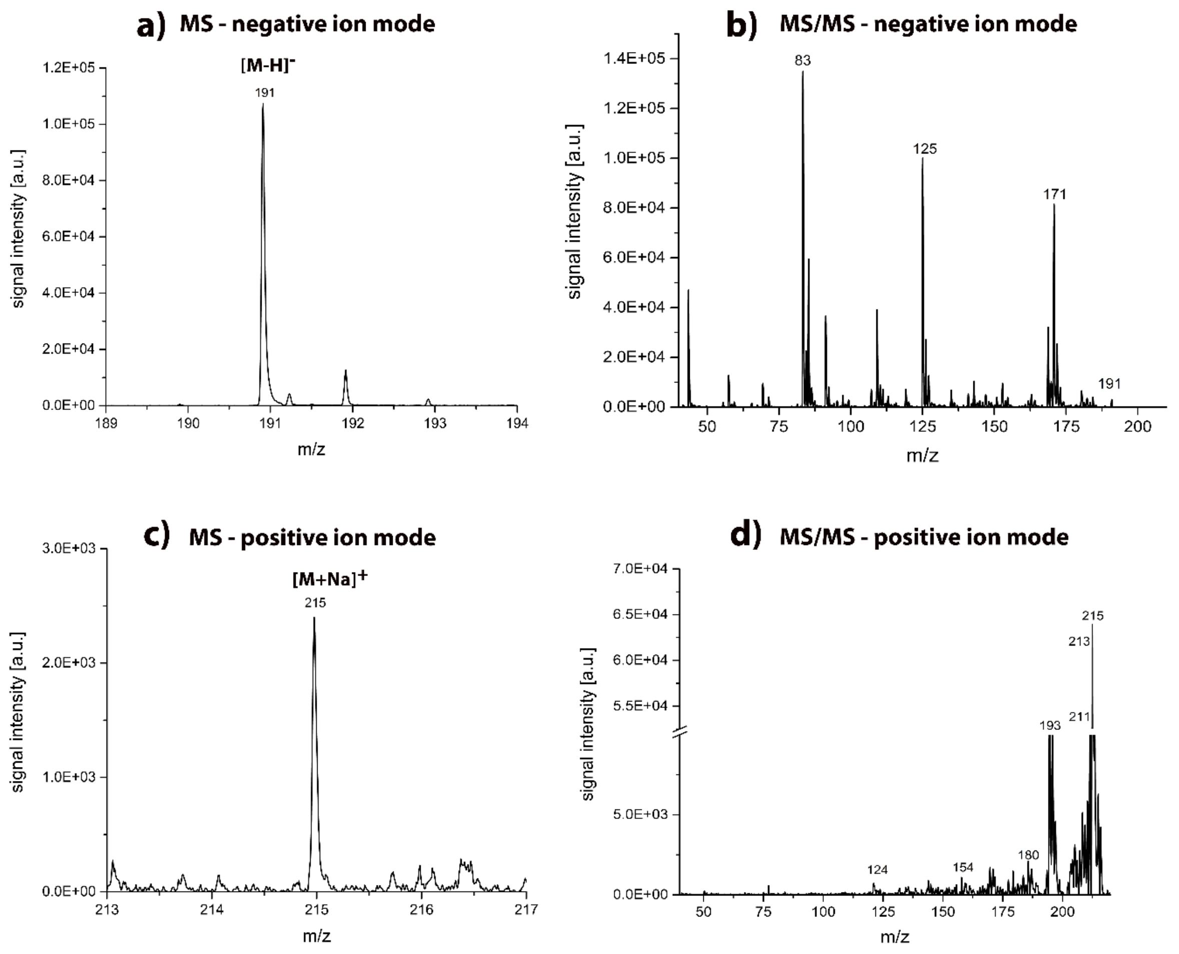

Fig. S5 LDI-TOF MS and MS/MS spectra of the reference standard quinic acid in the negative and positive ion mode, respectively

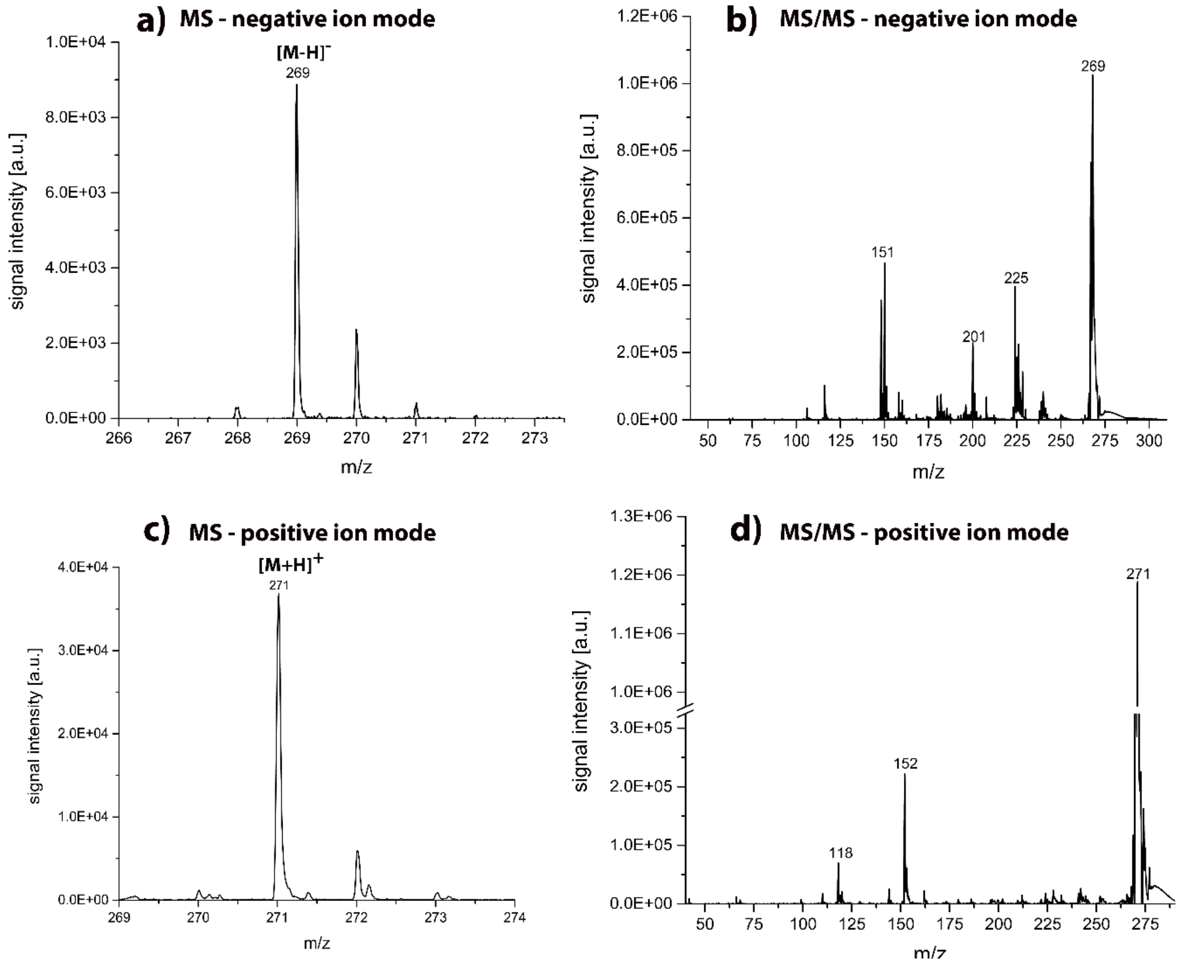

Fig. S6 LDI-TOF MS and MS/MS spectra of the reference standard apigenin in the negative and positive ion mode, respectively

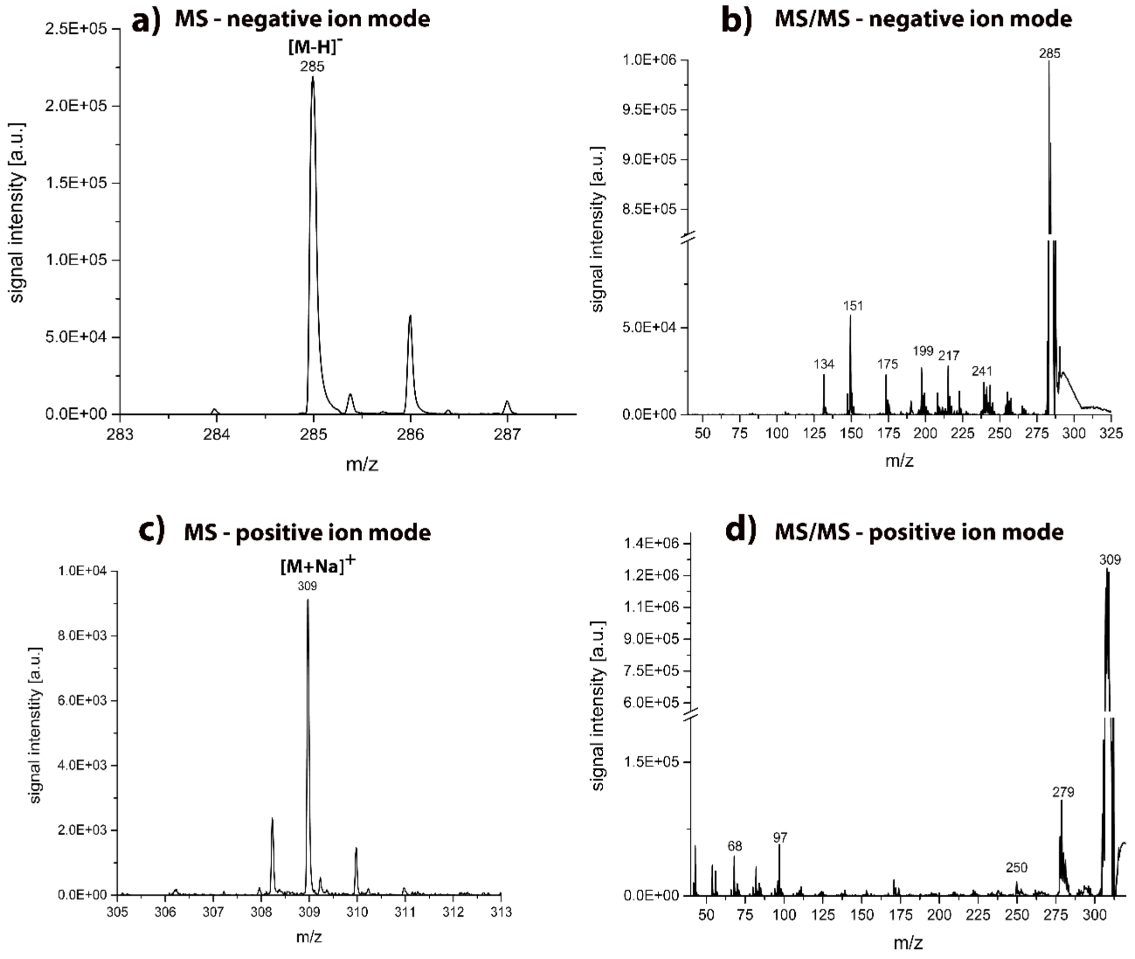

Fig. S7 LDI-TOF MS and MS/MS spectra of the reference standard luteolin in the negative and positive ion mode, respectively

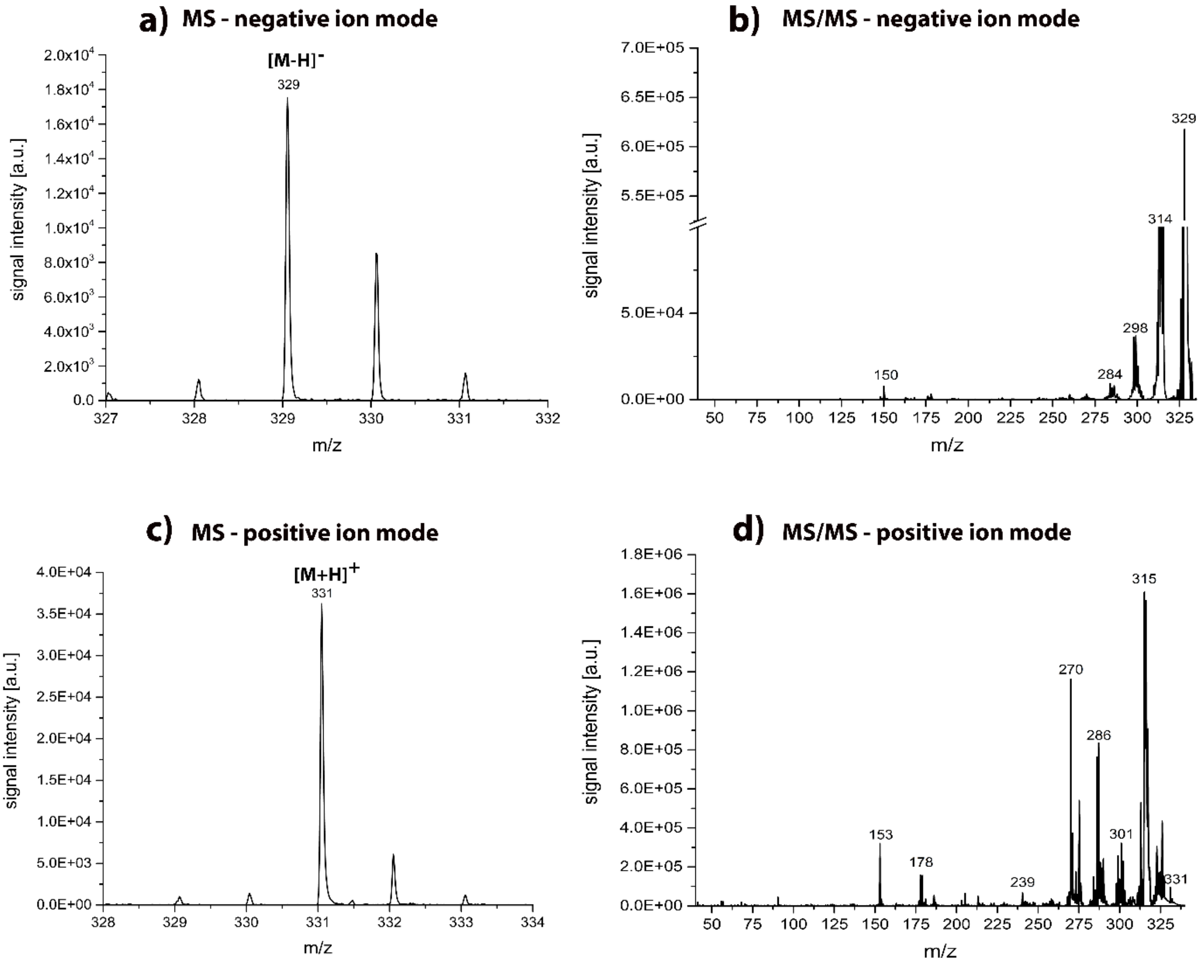

Fig. S8 LDI-TOF MS and MS/MS spectra of tricin, generated as in-source decay product from the reference standard tricin-5-glycoside, in the negative and positive ion mode, respectively

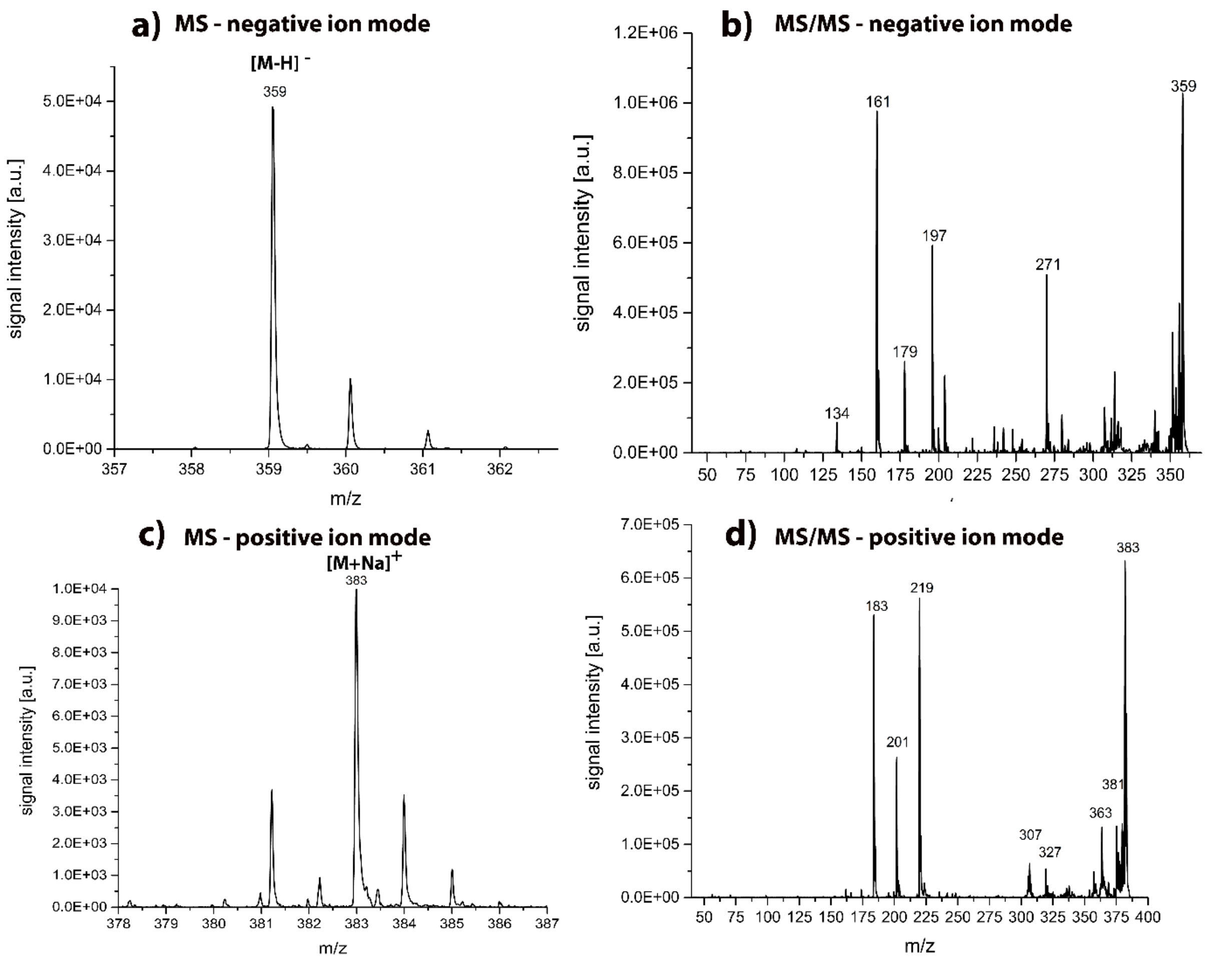

Fig. S9 LDI-TOF MS and MS/MS spectra of the reference standard rosmarinic acid in the negative and positive ion mode, respectively

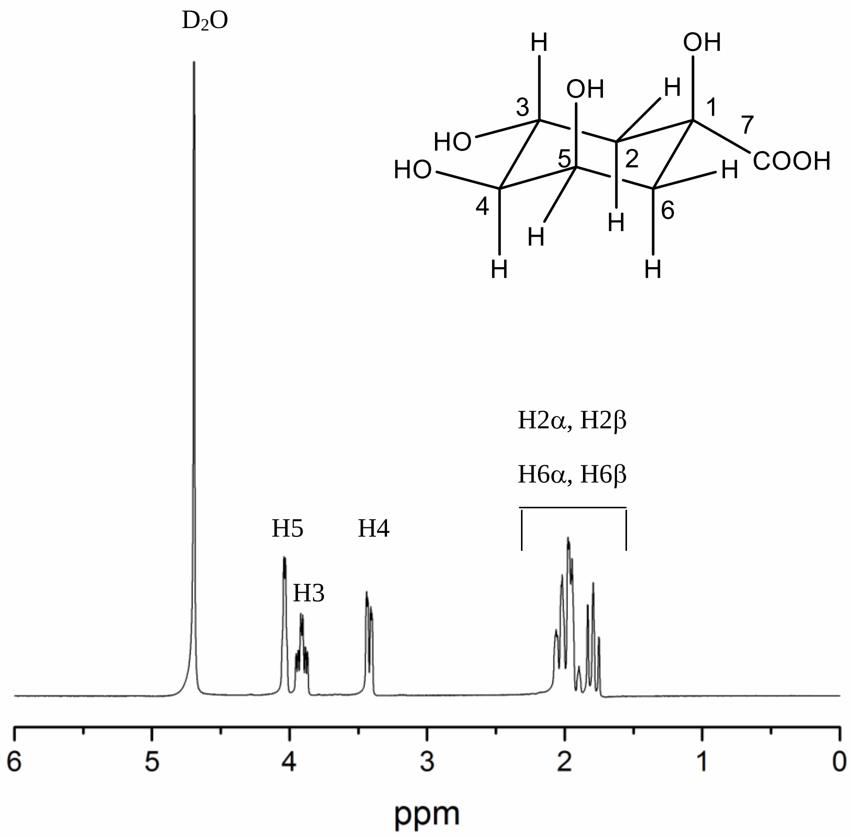

Fig. S10 ^{1}H NMR spectrum of the reference standard quinic acid

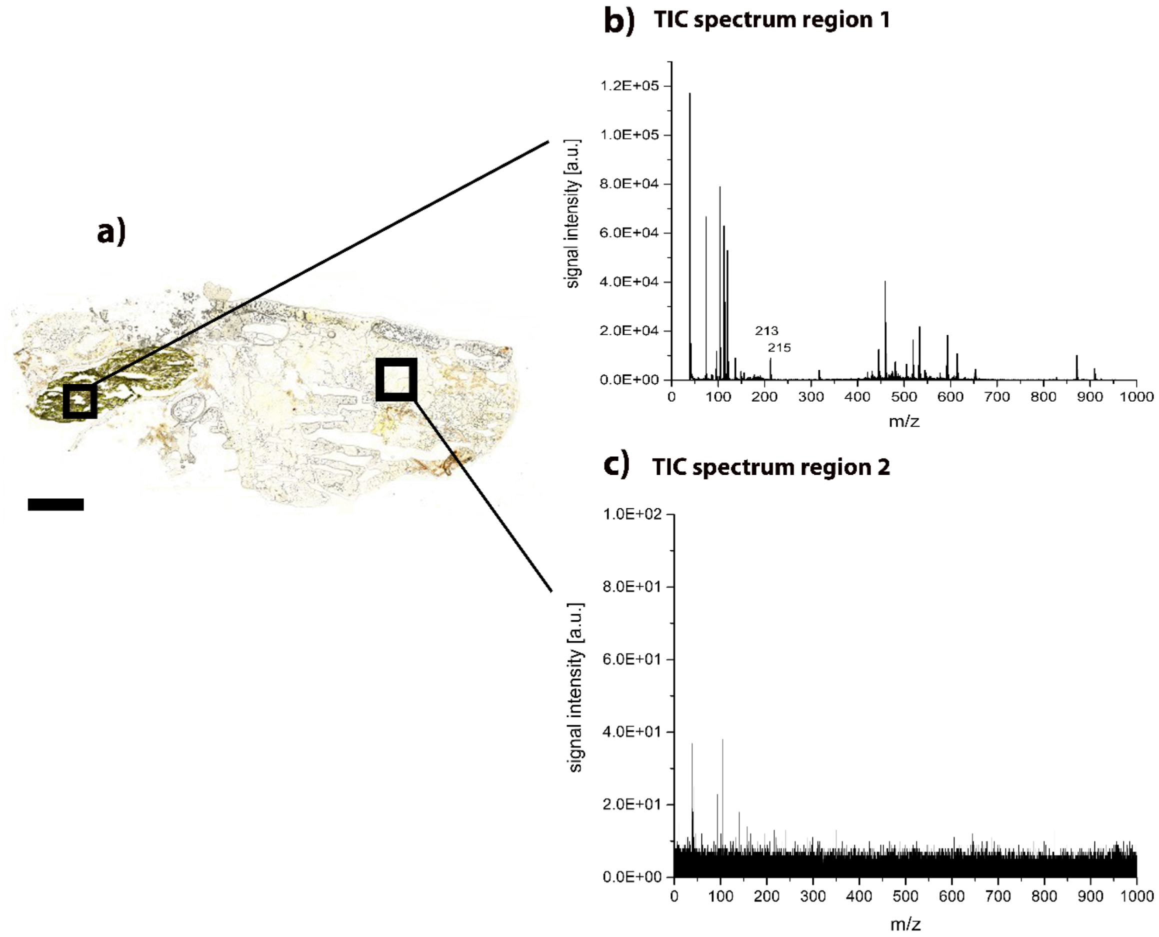

Fig. S11 LDI-TOF MSI dataset of the gastrointestinal tract of a female *Chorthippus dorsatus*. **a)** Optical image indicating two areas, from which TIC spectra were constructed, **b)** TIC spectrum from region 1, **c)** TIC spectrum from region 2. Scale bar 1 mm **a)**

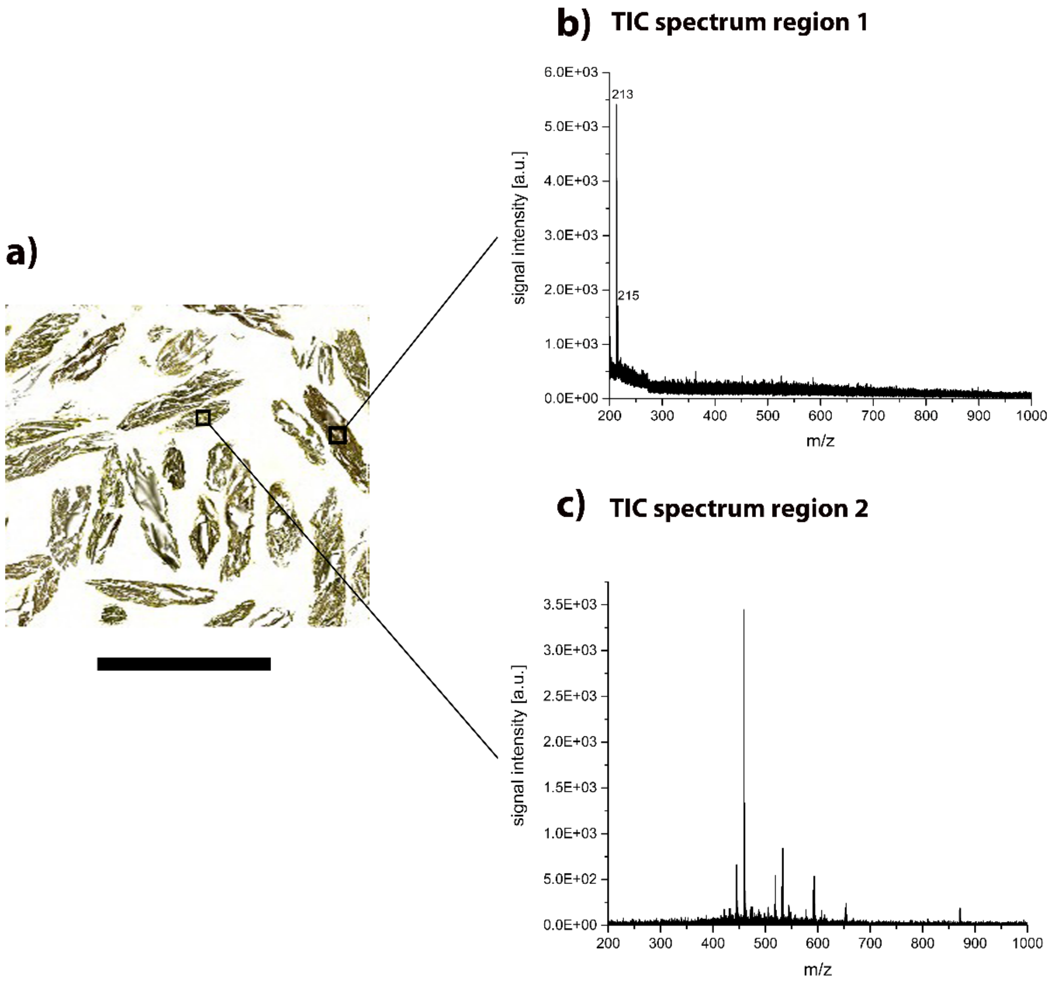

Fig. S12 LDI-TOF MSI dataset of excrements of *Chorthippus dorsatus*. **a)** Optical image indicating two areas, from which TIC spectra were constructed, **b)** TIC spectrum from region 1, **c)** TIC spectrum from region 2. Scale bar 5 mm **a)**
